# A Canine-Directed Chimeric Multi-Epitope Vaccine Induced Protective Immune Responses in BALB/c Mice Infected with *Leishmania infantum*

**DOI:** 10.3390/vaccines8030350

**Published:** 2020-06-30

**Authors:** Maria Agallou, Maritsa Margaroni, Stathis D. Kotsakis, Evdokia Karagouni

**Affiliations:** 1 Immunology of Infectious Diseases Laboratory, Department of Microbiology, Hellenic Pasteur Institute, 11521 Athens, Greece; mariaagallou@pasteur.gr (M.A.); mmargaroni@pasteur.gr (M.M.); 2 Laboratory of Bacteriology, Department of Microbiology, Hellenic Pasteur Institute, 11521 Athens, Greece; skotsakis@pasteur.gr

**Keywords:** reverse vaccinology, bioinformatics, multi-epitope vaccine, chimeric protein, T cells, adaptive immunity, innate immunity, long-term protection, visceral leishmaniasis

## Abstract

Leishmaniases are complex vector-borne diseases caused by intracellular parasites of the genus *Leishmania*. The visceral form of the disease affects both humans and canids in tropical, subtropical, and Mediterranean regions. One health approach has suggested that controlling zoonotic visceral leishmaniasis (ZVL) could have an impact on the reduction of the human incidence of visceral leishmaniasis (VL). Despite the fact that a preventive vaccination could help with leishmaniasis elimination, effective vaccines that are able to elicit protective immune responses are currently lacking. In the present study, we designed a chimeric multi-epitope protein composed of multiple CD8^+^ and CD4^+^ T cell epitopes which were obtained from six highly immunogenic proteins previously identified by an immunoproteomics approach, and the N-termini of the heparin-binding hemagglutinin (HBHA) of *Mycobacterium tuberculosis* served as an adjuvant. A preclinical evaluation of the candidate vaccine in BALB/c mice showed that when it was given along with the adjuvant Addavax it was able to induce strong immune responses. Cellular responses were dominated by the presence of central and effector multifunctional CD4^+^ and CD8^+^ T memory cells. Importantly, the vaccination reduced the parasite burden in both short-term and long-term vaccinated mice challenged with *Leishmania infantum*. Protection was characterized by the continuing presence of IFN-γ^+^TNFα^+^-producing CD8^+^ and CD4^+^ T cells and increased NO levels. The depletion of CD8^+^ T cells in short-term vaccinated mice conferred a significant loss of protection in both target organs of the parasite, indicating a significant involvement of this population in the protection against *L. infantum* challenge. Thus, the overall data could be considered to be a proof-of-concept that the design of efficacious T cell vaccines with the help of reverse vaccinology approaches is possible.

## 1. Introduction

The major goal of vaccines against obligate intracellular pathogens, such as *Leishmania*, is to activate the immune system towards strong immune responses, mainly composed of CD4^+^ and CD8^+^ T cells. These cell populations are critical for disease control and long-term protection through the production of IFN-γ and cytotoxic effects [1]. However, effective vaccines against these pathogens are currently lacking, since elicitation of the desirable cell-mediated response by vaccination is more difficult than obtaining humoral responses mainly due to the antigenic complexity of the pathogen [2]. *Leishmania* parasites are transmitted by the bite of female sand flies and the establishment of an infection through a host immune response evasion causing leishmaniases, an important group of neglected tropical diseases [3]. The diseases have multiple clinical forms depending on the different species of the parasite, the genetic makeup of the host, and the host–parasite interaction [4]. Among them, visceral leishmaniasis (VL), caused by *L. donovani* and *L. infantum*, is the most severe systemic form of the leishmaniasis disease in humans. It is characterized by fever, cachexia, hepatosplenomegaly, hypergammaglobulinemia, and pancytopenia and is fatal if left untreated. Its estimated disease burden places it second in mortality and fourth in morbidity among the tropical infectious diseases [5]. Moreover, in southern Europe and the Mediterranean region, dogs serve as effective reservoirs by infecting phlebotomine sand fly vectors. The result is a dynamic spectrum of naturally infected dogs ranging from resistant, asymptomatic animals to those with severe disease [6]. Until now, the control of VL has been focused on chemotherapy of affected individuals and control of infected animals and vectors. However, the currently accessible drugs are toxic, have serious side effects, and are related to an increased incidence of drug resistance, making the development of a safe and effective vaccine an urgent need [7,8]. 

Initially, development of a vaccine against *Leishmania* infection involved the inoculation of live or autoclaved parasites, a practice that has been abandoned due to safety concerns [9,10]. Nowadays, numerous vaccine constructs have been proposed including killed or live attenuated strains of the parasites, crude extracts, or purified recombinant antigens [11]. However, evidence from mouse models, suggest that although the new vaccines represent improvements over leishmanization, few of them are tested in human clinical trials. Moreover, only three vaccines are commercially available against canine leishmaniasis [12]. On the one hand, these vaccines that contain antigens recognized by symptomatic infected dogs, in many cases, fail to control infection. On the other hand, asymptomatic infections that resolve without manifesting disease are controlled through induction of an effective antigen-specific cell-mediated immune response [13]. Specifically, it has been shown that T cells from asymptomatic infected and cured individuals or dogs respond to *Leishmania* antigens by producing IFN-γ [14]. 

In a previous study by our group, we conducted a comparative immunoproteomics analysis of *L*. *infantum* protein extracts using sera from asymptomatic and symptomatic dogs naturally infected with the parasite in order to identify molecules that are recognized by antibodies found exclusively in asymptomatic hosts [15]. The experimental evidence indicated mitochondrial precursor chaperonin HSP60 (Cpn60), dihydrolipoamide dehydrogenase (Gcvl-2), enolase (Eno), cyclophilin 2 (CyP2), cyclophilin 40 (CyP40), and a hypothetical protein (HyP) as potential vaccine antigens based on their exclusive recognition by asymptomatic dogs’ antibodies and their high content in MHC class I and MHC class II epitopes [15]. Interestingly, some of these proteins were highly conserved among *Leishmania* spp. [16] and were shown to play a role in parasite viability and resistance against treatment [17,18,19,20,21], whereas others, i.e., enolase, also have a proven capacity to induce protective T_H_1 immune responses in the experimental models of VL when used as a vaccine [22,23,24]. 

Increased knowledge and improved understanding of pathogen variability and diversity of the human immune system suggest that the induction of protective immune responses can be achieved through development of multistage multi-epitope vaccines. These vaccines should include cytotoxic T lymphocyte (CTL) and helper T lymphocyte (HTL) epitopes, in order to generate the best combined protective T cell response [25]. Improved immunoinformatics approaches further allow the prediction of antigenic epitopes that have the ability to bind to a broad range of MHC class I and class II alleles, circumventing definite haplotype immune responses [26]. Despite the several advantages of multi-epitope vaccines, they are characterized by poor immunogenicity. For this reason, the epitope-based vaccines are regularly fused with built-in microbial adjuvant proteins, especially toll-like receptors (TLR) ligands that can polarize CD4^+^ T cells and induce CTL responses [27]. Recently, heparin-binding hemagglutinin (HBHA), a surface component of *Mycobacterium tuberculosis*, has been investigated for its strong immune potential. Specifically, it has been shown that HBHA could stimulate migration of DCs and promoted their maturation in a TLR4-dependent manner [28]. Subcutaneous immunization of mice with the N-terminal domain of HBHA was sufficient to trigger humoral and cellular immune responses [29]. In addition, when this domain was used as an adjuvant in a multi-epitope vaccine against cancer, it induced a strong T_H_1 cell immune response [30,31]. 

In the present study, we designed a novel multi-epitope protein vaccine against *Leishmania* parasites by implementing several bioinformatic tools. This vaccine consisted of HTL and CTL epitopes extracted from the previously identified immunodominant proteins Cpn60, Gcvl-2, Eno, CyP2, CyP40, and the hypothetical protein in order to stimulate cellular immunity. These epitopes were linked together with the N-terminal domain of *M*. *tuberculosis* HBHA as an adjuvant to resolve the low immunogenicity issue of the multi-epitope domain. Structural conformation, stability, and interaction studies of the proposed vaccine with TLR4 were carried out in silico. Moreover, the potential of the developed vaccine for inducing protective immune responses against *L*. *infantum* was evaluated in vitro and in vivo in the experimental model of VL in BALB/c mice. 

## 2. Materials and Methods 

### 2.1. In Silico Prediction of Helper T Lymphocyte (HTL) and Cytotoxic T Lymphocyte (CTL) Epitopes

The antigens selected for this study, i.e., cyclophilin 2 (XP_001463094), cyclophilin 40 (XP_001469283), enolase (XP_001468063), dihydrolipoamide dehydrogenase (XP_001468025), mitochondrial chaperonin HSP60 (XP_001467869), and a hypothetical protein (XP_001463461) of *L. infantum* parasite were previously characterized as candidate vaccine antigens, due to their exclusive recognition by serum antibodies obtained from asymptomatic dogs infected with *L. infantum* [15]. Their amino acid sequences were all retrieved from the NCBI (National Centre for Biotechnology Information) protein database (http://www.ncbi.nlm.nih.gov/protein/) in FASTA format. In the first step of the analysis, amino acid sequences were screened individually for the best binding CTL epitopes against murine H2-Dd, H2-Kd, and H2-Ld alleles using the IEDB MHCI (http://tools.iedb.org/mhci/), SYFPEITHI (http://www.syfpeithi.de/bin/MHCServer.dll/EpitopePrediction.htm) and NetCTLpan (http://www.cbs.dtu.dk/services/NetCTLpan/) online servers. Prediction threshold values of percentile rank ≤10.0 for IEDB, ≥20 for SYFPEITHI and ≤0.75 for NETCTLpan1.1 were set for epitope identification. For the prediction of HTL epitopes against MHC class II molecules H2-IAd and H2-IEd alleles, the IEDB MHCII (http://tools.iedb.org/mhcii/), SYFPEITHI, and NetMHCII 2.2 (http://www.cbs.dtu.dk/services/NetMHCII-2.2/) servers were employed. Prediction threshold values of percentile rank ≤20.0 for IEDB and ≥20 for SYFPEITHI, whereas in NetMHCII an IC50 value ≤50 nM for high binders and an IC50 value ≤500 nM for weak binders were set for epitope identification. The peptides that were predicted by at least two algorithms were selected for a second step of analysis. For this, the selected peptides were analyzed using the IEDB server against the HLA class I and II alleles reference sets that provided >97% and >99% population coverage, respectively. To screen out only non-human and non-mouse homologues epitopes, the obtained epitopes were subjected to BLASTp analysis (https://blast.ncbi.nlm.nih.gov/Blast.cgi) against the host proteomes, i.e., Homo sapiens (taxid: 9606) and Mus musculus (taxid: 10090). Epitopes having >80% sequence identity were extracted from the study. Then, the conservancy of the obtained CTL and HTL epitopes within a given protein sequence of 5 different strains of *Leishmania* parasites (*L.infantum*, *L. donovani*, *L. major*, *L. mexicana*, and *L. braziliensis*) was determined by applying the IEDB conservancy tool (http://tools.immuneepitope.org/tools/conservancy/). For this purpose, the sequence identity parameters were set to default. Moreover, the population coverage of individual peptide epitopes was also calculated using the IEDB population coverage analysis tool (http://tools.iedb.org/tools/population/iedb_input). Finally, the selected peptides were synthesized by GeneCust (Ellange, Luxenbourg) with a purity >95%, as determined by HPLC. The synthetic peptides were dissolved in DMSO and stored in aliquots, in −80 °C until use. For ex vivo stimulation assays, the following two peptide pools were used: CTL pool consisted of the six selected 9-mer peptides and HTL pool consisted of the six selected 15-mer peptides.

### 2.2. Design and In Silico Evaluation of the Multi-Epitope Vaccine 

The selected CTL (CyP2_126–134_, CyP40_232–240_, Gcvl-2_439–447_, Enol_186–194_, Cpn60_38–46_, and Hyp_169–177_) and HTL (CyP2_173–187_, CyP40_332–340_, Gcvl-2_19–33_, Enol_111–125_, Cpn60_27–41_, and Hyp_57–71_) epitopes were lined up together to construct a multi-epitope (LiChimera) vaccine. Specifically, the C-termini of the CTL epitopes were flanked together by AAY linker, whereas the GPGPG linker was used for HTL epitopes. To increase the vaccine’s immunogenicity, the 1–159 amino acid sequence of *M. tuberculosis* (strain ATCC 25618 / H37Rv) HBHA (ZP_07011362.1) was linked to the N-terminal of the vaccine construct via the EAAAK linker. The physicochemical properties of the multi-epitope vaccine including molecular weight (Mw), theoretical pI, instability index, aliphatic index, estimated half-life, and grand average of hydropathicity (GRAVY) were evaluated using the ProtParam tool (https://web.expasy.org/protparam/). The multi-epitope vaccine construct’s allergenicity prediction was conducted by employing AlgPred, Allerdictor, and AllergenFP tools and its antigenicity was predicted by employing the VaxiJen v2.0 and ANTIGENpro servers. The SOLpro server (http://scratch.proteomics.ics.uci.edu/) was applied for predicting protein’s solubility in *E. coli*. 

### 2.3. Computational Structural and Docking Analyses of Heparin-Binding Hemagglutinin (HBHA) 

Three-dimensional complexes of the coiled-coil domain of *M. tuberculosis* HBHA and the LiChimera protein bound to the TLR4/MD2 complex were built and simulated through molecular dynamics in order to assess any likely effects of the multi-epitope domain on binding. 

Three-dimensional structures of the two proteins were built through homology modeling using MODELLER v9.21 as follows [32]. The *M. tuberculosis* HBHA (Protein ID: WP_070896710.1) homologues with solved structures were identified using an HHpred (https://toolkit.tuebingen.mpg.de/tools/hhpred) search against the PDB_mmCIF70 database [33]. The coiled-coil domain of HBHA yielded high quality alignment with the NMR structure of the Apoliphorin-III from *Manduca sexta* (PDB ID: 1EQ1) that was selected as the reference for building the model. Portions of the protein predicted to form α-helices were constrained to adopt this structure. A thorough VTFM optimization was applied with maximum iterations set to 1000 and the models were refined using the “very slow” molecular dynamic (MD) simulated annealing setting. Optimization was repeated for 10 cycles and the objective energy function cut-off was set to 5 × 10^6^. Models exhibiting the lowest probability density function values and with no knots present were selected for further refinement through 20 ns MD simulations in GROMACS 2016.3 [34] using the atom parameters of the AMBER99-ILDN force field. From the stable phase of each simulation, an average structure was calculated and minimized. The model with the lowest potential energy was selected for subsequent analyses. The three-dimensional structure of the LiChimera protein was built using the above structure as the reference for the HBHA moiety. The residues 160 to 353, comprising the multi-epitope domain of the protein, were subjected to an HHpred search and fragments from the following structures were used as templates: 178–225: Putative carboxymuconolactone decarboxylase *Burkholderia xenovorans* (PDB ID: 2QEU; 82-129), 230–277: *Trypanosoma*, mitoribosomal subunit (PDB ID: 6HIX; 173–220), 279–303: Dihydrolipoamide dehydrogenase from yeast (PDB ID: 1V59; 12–45). The predicted secondary structure for the non-HBHA domain of the protein was used to apply the appropriate constraints. Models were generated and refined as above.

Docking of HBHA and LiChimera on the TLR4/MD2 complex was performed using HADDOCK2.2 (https://milou.science.uu.nl/services/HADDOCK2.2) [35]. The coordinates for the TLR4/MD2 complex were extracted from the TLR4/MD2/lipid IVa crystal structure from *Mus musculus* (PDB ID: 3VQ1) [36]. Missing atoms were added using MODELLER and the dimer form of the TLR4/MD2 was minimized in vacuo in GROMACS using the AMBER99-ILDN force field. Assignment of active residues for the receptor was based on previous data regarding the likely binding of the HBHA onto the MD2 subunit of the complex [30]. The surface accessible surface area (SASA) for every residue in the complex was calculated using the VMD software. The MD2 surface residues exhibiting SASA values higher that 70 Å^2^ were selected as the active residues. The TLR4/MD2 residues located within 5 Å of the above active residues and exhibiting a SASA greater than 40 Å^2^ were selected as the passive residues. Active and passive residues in HBHA and LiChimera were similarly selected. For each docking simulation, the first structure of the cluster with the lowest Z-score was considered to be the best docking solution and was selected for the MD simulations in GROMACS.

Complexes were minimized in vacuo using 10,000 steps of steepest descent (SD) and 2500 steps of conjugated gradient (CG) energy minimization. Then, each system was embedded in a cubic box using a margin of 0.1 nm and solvated in TIP3P water model. Charges were neutralized using Na+ and Cl- ions at a concentration of 0.15 M. Then, the systems were minimized using 5000 steps of steepest descent energy minimization. Equilibration was carried out by an initial simulation in constant volume and temperature at 300 K (NVT) with protein heavy atoms being position restrained using a force of 1000 KJ/mol/nm for 100 ps. A time step of 2 fs was used to integrate the Newton’s equations of motion and the length of all bonds was constrained using the LINCS algorithm. The Verlet cut-off scheme was used for non-bonded interactions and the particle mesh Ewald method was used to treat long-range electrostatic interactions. Initial velocities were generated at 300 K from a Maxwell distribution. A second position-restrained equilibration was performed for 50 ps using a constant pressure of 1 bar and a constant temperature of 300 K (NPT) with initial velocities being obtained from the last step of the previous NVT simulation. The unrestrained production simulation was performed in an NPT ensemble as above for 100 ns. Stability of the simulated complexes was assessed using the root mean square deviation (RMSD) of backbone protein atoms’ position during the trajectories using as a reference the starting structure, as well as the radius of gyration of protein atoms (Rg) as a measure of complex compactness. Cluster analysis was performed for the stable phase of each simulation using the GROMOS clustering algorithm and a cut-off of 1 Å for backbone atoms. For the most frequent cluster, an average structure was calculated and minimized using SD energy minimization. Binding energies of the HBHA and LiChimera proteins on the TLR4-MD2 complex were estimated during the trajectories using the molecular mechanics Poison–Boltzmann surface area (MMPBSA) method as implemented in the g_mmpbsa program using GROMACS and APBS packages [37]. Binding energies were calculated using the python script MmPbSaStat.py and the contribution of each amino acid in binding by the script MmPbSaDecomp.py included in the g_mmpbsa program.

### 2.4. Expression and Purification of the Chimeric Multi-Epitope Vaccine 

Subsequently, the 1068 bp nucleotide sequence which was designed to encode the vaccine protein was codon optimized for translation by the E. coli K12 host using JCat software (http://www.jcat.de/). The optimized sequence was reversed and NdeI and XhoI restriction sites were added. In silico cloning was conducted using the SnapGene restriction cloning tool and the pET-30a(+) plasmid vector was used to insert the optimized codon sequence. The multi-epitope vaccine was commercially synthesized by GeneCust. Specifically, the chimeric construct was used to transform *E. coli* BL21 (DE3) and protein expression was induced with 1 mM isopropyl-β-D-thiogalactopyranoside (IPTG) for 3 h. The recombinant chimeric protein was purified with a Ni-nitrilotriacetic acid (NTA) affinity column and the expressed protein was analyzed by SDS-PAGE under denaturing conditions of 15% polyacrylamide gels. Finally, endotoxin was removed with the Endotoxin Removal Beads and the amount of the residual endotoxin was less than 0.1 EU/μg of purified protein as assessed using a Limulus Amebocyte Lyase (LAL) kit.

### 2.5. Canine Peripheral Blood Mononuclear Cells (PBMCs) Lymphoproliferation

The collection of peripheral blood mononuclear cells (PBMCs) was conducted with National Law 160/01 (FEK 64/A/91), which adheres to the European Directive 86/609/EEC on the approximation of laws, regulations, and administrative provisions of the Member States regarding the protection of animals used for experimental and other scientific purposes. Clinical examination, diagnosis, and treatment were conducted by an experienced veterinarian of the diagnostic department of Hellenic Pasteur Institute. The dogs were examined by observing the typical clinical signs of canine visceral leishmaniasis (CVL) (ocular and/or skin lesions, onychogryphosis, progressive weight loss, muscular atrophy, epistaxis, apathy, and generalized lymphadenomegaly, among others) and were subjected to serological (immunofluorescence antibody tests (IFAT and enzyme-linked immunosorbent assays (ELISA)) and molecular (real-time PCR) diagnostic tests to verify infection by *L. infantum*. Healthy dogs did not present clinical signs or clinicopathological abnormalities and were characterized as *Leishmania* negative based on serological and molecular diagnostic tests. Symptomatic dogs were characterized as animals with IFAT titers of greater than 1:200, positive ELISA and PCR results, and more than 2 clinical symptoms and these dogs were subjected to anti-leishmanial treatment. Canine peripheral blood mononuclear cells (PBMCs) were isolated from heparinized blood from healthy dogs or infected dogs at the end of their treatment by density centrifugation (Lymphosep, Biowest). The PBMCs (2 × 10^5^ cells/well) were cultured, in triplicate, in 96-well round bottom plate. The cells were stimulated with LiChimera (10 µg/mL) or soluble *Leishmania* antigen (SLA) (10 µg/mL) for 5 days at 37 °C and 5% CO_2_. PBMCs incubated in medium alone served as the negative control of proliferation, whereas PBMCs incubated in the presence of ConA (10 µg/mL) served as the positive control. Cell proliferation was measured, during the last 18 h of culture, by measuring [^3^H]-TdR (Perkin Elmer, Boston, MA, USA) incorporation with the help of a microplate scintillation β-counter (Microbeta Trilux, Wallac, Turcu, Finland). The results were obtained as counts per minute (cpm).

### 2.6. Mice 

Studies were performed with 6–8-week-old female BALB/c mice. Animals were housed in SPF (specific pathogens-free) conditions of Hellenic Pasteur Institute at room temperature 22 ± 2 °C, relative humidity 40–70%, and 12 hours light/12 hours dark cycle. All procedures complied with PD 56/2013 and European Directive 2010/63/EU, welfare and ethical use of laboratory animals based on 3+1R and the guidelines of PREPARE (Planning Research and Experimental Procedures on Animals: Recommendations for Excellence), ARRIVEs (Animal Research: Reporting In Vivo Experiments), and ARRIGE (Association for Responsible Research and Innovation in Genome Editing). The experimental protocol was licensed under the registered code 6381/11-12-2017, by the Official Veterinary Authorities of Attiki’s Prefecture. 

### 2.7. Parasites and Soluble Leishmania Antigen Production

The *L. infantum* (MHOM/GR/2001/GH8) parasites were obtained from the infected BALB/c mice and were cultured in vitro at 26 °C in complete medium. The complete medium consisted of RPMI-1640 (Biowest) supplemented with 2 mM L-glutamine, 10 mM HEPES, 24 mM NaHCO3, 100 U/mL penicillin, 10 µg/mL streptomycin, and 10% (v/v) heat-inactivated fetal bovine serum (Biowest). 

The soluble *Leishmania* antigen (SLA) was prepared according to a previously described protocol from stationary phase *L. infantum* promastigotes [38]. The SLA was collected and stored at −80 °C until use.

### 2.8. Immunization and Challenge of Mice

The BALB/c mice were immunized intramuscularly on weeks 0 and 2 with 10 µg of LiChimera alone or adjuvanted with Addavax mixed 1:1 (InvivoGen, Toulouse, France) in a total volume of 80 µL (40 µL/quadricep). The control mice received an equal volume of sterile PBS or Addavax. Protective efficacy was evaluated in two separate experiments. In the first experiment, all mouse groups were challenged via tail vein with 10^7^ stationary-phase *L. infantum* promastigotes 2 weeks post-boosting vaccination and parasite load was measured in spleens and liver 8 weeks post challenge. The second experiment aimed at characterizing the protective efficacy promoted by vaccine-induced memory responses and in this case all mouse groups were challenged 12 weeks post-boosting vaccination. Similarly, parasite load was estimated 8 weeks post challenge. In T cell subset depletion experiments, mice were vaccinated with LiChimera adjuvanted with Addavax, as described above, and then mice were injected intraperitoneally with rat anti-CD8 (clone 53-6.7, Biolegend) monoclonal antibody or isotype control IgG (clone RTK2758, Biolegend) at 500 µg/mouse, given on days -1 and +2 relative to the day of parasite challenge. The efficiency of T cell depletion was assessed by flow cytometry analysis of splenocytes on day 5 using naïve mice.

### 2.9. Determination of Live Parasite Burden by Limited Dilution Analysis (LDA)

The parasite burden in spleen and liver was determined by limited dilution analysis (LDA). Briefly, a portion of liver or spleen that had been previously weighed was homogenized in Schneider’s Insect Medium and resuspended at a final concentration of 1 mg/mL in Schneider’s medium supplemented with 20% FBS. Serial dilutions (2-fold) of the tissue homogenates were incubated at 26 °C, for 7 days. The presence of viable and motile promastigotes was examined at a 3-day interval. The reciprocal of the highest dilution that was positive for parasites was the parasite concentration per milligram of tissue.

### 2.10. Measurement of Delayed-Type Hypersensitivity (DTH)

The delayed-type hypersensitivity (DTH) response was evaluated as an index of cell-mediated immune response. Mice from all groups were intradermally inoculated in footpad with 50 μg of SLA in a total volume of 30 μL of PBS, 10 days post booster vaccination or 8 weeks post *L. infantum* challenge in long-term vaccinated mice. The DTH was evaluated by measuring the difference in footpad swelling 24 h following inoculation with that of the control PBS injected footpad using a dial caliper (Kori Seiki MFG Ltd, Tokyo, Japan).

### 2.11. Detection of LiChimera- and Parasite-Specific IgG, IgG1, and IgG2a Antibodies

LiChimera-specific IgG, IgG1, and IgG2a production was determined through ELISA in sera collected from all experimental mice groups at predetermined time points. Sera samples were added at a dilution of 1:400 for 90 min in 96-well microtiter plates pre-coated with 2 μg/mL of LiChimera. After that, HRP-conjugated anti-mouse IgG (1:5000 dilution) (Thermo Scientific) or biotinylated anti-mouse IgG1 (1 μg/mL) and IgG2a (250 ng/mL) (both obtained from AbD Serotec, Oxford, UK) were added for 1 h, at 37 °C. In the case of biotinylated antibodies streptavidin-HRP at a dilution of 1:5000 was added and samples were incubated for another 1 h, at 37 °C. The enzyme-labeled complexes were detected by reaction with TMB substrate. The absorbance was measured at 450 nm using an ELISA microplate spectrophotometer (MRX). In some cases, detection of the parasite-specific IgG1 and IgG2a antibodies were also conducted according to a previously published protocol for mice [39]. 

### 2.12. LiChimera- and Parasite-Specific Proliferation Assay

Spleen cells were isolated from vaccinated and non-vaccinated mice at 2 weeks post-booster vaccination as well as 8 weeks post challenge for the evaluation of antigen-specific proliferation. For this purpose, cells were cultured in 96-well round-bottom plates (2 × 10^5^ cells/200µL/well) in the presence of LiChimera (2.5 µg/mL) or SLA (12.5 μg/mL) for 96 h, at 37 °C, and 5% CO_2_. Con A (6 µg/mL) stimulated spleen cells served as a positive control for proliferation and spleen cells cultured in medium alone served as a negative control. Cell proliferation was measured by [^3^H]-TdR incorporation as described above incorporation and the results were presented as stimulation index based on the formula: S.I. = cpm measured in lymphocytes in the presence of antigen or mitogen/cpm measured in lymphocytes in medium alone.

### 2.13. Bone Marrow-Derived Dendritic Cell (BMDC) Differentiation and Evaluation of Their Maturation after Sensitization with Multi-Epitope Vaccine

The bone marrow-derived dendritic cells (BMDCs) were generated from the isolated bone marrow derived cells, as previously described [40]. On days 6–7, non-adherent cells were harvested and BMDC maturation induced by LiChimera was assessed by flow cytometry. Specifically, BMDCs were seeded in 24-well plates (1 × 10^6^ cells/mL) and cultured in the presence of LiChimera (2.5 µg/mL) or lipopolysaccharide (LPS) (1 µg/mL) as a positive control of maturation for 24 h. The BMDCs cultured in medium alone were considered to be immature cells. At the end of incubation, cells were washed with PBS containing 3% (v/v) FBS (FACS buffer), and then were labeled with PE-conjugated anti-mouse CD11c (clone HL3), R-PE-conjugated anti-mouse CD40 (clone 3/23), CD80 (clone 16-10A1), and MHCI (clone SF1-1.1) (all used in 1:100 dilution), or MHCII (clone 2G9; 1:200 dilution) monoclonal antibodies for 30 min, at 4 °C, in the dark. All antibodies were purchased from BD Biosciences (Erembodegem, Belgium). Then, cells were fixed with 2% PFA for 20 min, at 4 °C, in the dark, followed by a wash with FACS buffer. The BMDC maturation analysis was conducted using a FACSCalibur system (Becton-Dickinson, San Jose, CA, USA) running CellQuest software. Data were analyzed using FlowJo software version 10.0 (Tree Star Inc., Ashland, OR, USA).

### 2.14. Antigen-Presenting Capacity of LiChimera-Pulsed BMDCs 

The BMDCs were generated from the isolated bone marrow-derived cells, as described above. On day 7, non-adherent cells were harvested and BMDCs were cultured for 24 h in the presence of LiChimera (2.5 µg/mL). The BMDCs cultured in medium alone served as the control. Excess stimulators were washed out and 2 × 10^4^ BMDCs were cocultured with 1 × 10^5^ CD4^+^ or CD8^+^ T cells that were isolated from spleens of mice vaccinated with either LiChimera or LiChimera adjuvanted with Addavax, by magnetic beads sorting (Invitrogen, Carlsabad, USA). Cells were plated in a 96-well round-bottom plate and T cell proliferation was assayed on day 4, by [^3^H]-TdR incorporation, as described above in the cell proliferation assay.

### 2.15. Multiparametric Flow Cytometric Analysis

Spleen cells isolated at 2 weeks post booster immunization, as well as 8 weeks post *L. infantum* challenge, were plated at 1 × 10^6^ cells/well in a 24-well flat-bottom plate and stimulated with 5 µg/mL LiChimera or peptide pools of 9-mer peptides (6 peptides, 2 µg/mL each) or 15-mer peptides (6 peptides, 2 µg/mL) for 12 h. Subsequently, they were incubated for 4 h, at 37 °C, following the addition of 10 µg/mL brefeldin A (Cayman, Michigan, USA). Then, cells were washed in FACS buffer and stained for 30 min at 4 °C, in the dark, for surface markers using anti-CD4-PerCP-Cy5.5 (clone RM4-5), anti-CD8-PerCP-Cy5.5 (clone 53-6.7), anti-CD44-APC (clone IM7), and anti-CD62L-PE (clone MEL-14) mAbs at a dilution of 1:100. Then, cells were washed in FACS buffer, and fixed with 2% paraformaldehyde for 20 min. For intracellular staining, cells were permeabilized with FACS buffer containing 0.1% saponin and stained intracellularly for 30 min at 4 °C, in the dark, using anti-IFN-γ-PE (clone XMG1.2), anti-TNF-α-FITC (clone MP6-XT22), and anti-IL-2-APC (clone JES6-5H4) mAbs. Subsequently, cells were washed, resuspended in PBS, and then analyzed using a FACSCalibur system (Becton-Dickinson, San Jose, CA, USA) running CellQuest software. Data were analyzed using FlowJo software version 10.0 (Tree Star Inc., Ashland, OR, USA). All antibodies used were obtained from Biolegend.

### 2.16. Bone Marrow-Derived Macrophages (BMMs) Generation and In Vitro Infection with L. infantum

The bone marrow-derived macrophages (BMMs) were generated from bone marrow derived cells isolated two weeks post booster vaccination from vaccinated and non-vaccinated mice. Briefly, bone marrow from femurs and tibiae was flushed into RPMI and the cells obtained were cultured in complete RPMI supplemented with 20 ng/mL recombinant murine GM-CSF. On day 3, fresh medium containing GM-CSF was added. Cells were allowed to differentiate into macrophages (attached cells) for a total of 6 days. Then, cells were harvested by removing the supernatant and the help of a cell-scraper. BMMs were resuspended in complete RPMI and allowed to adhere to coverslips seeded in a 24-well plate at a density of 1 × 10^6^/mL, for 2 h. Non-adherent cells were removed by gentle washing with warm PBS and macrophages were infected with stationary phase *L. infantum* promastigotes at a ratio 10:1, for 3 h. The non-phagocytosed parasites were removed by warm sterile PBS and BMMs were further incubated for 72 h. Finally, culture supernatants were collected for NO determination. For the determination of intracellular parasite load, BMMs were fixed in methanol followed by Giemsa staining at 24, 48, and 72 h post infection. 

### 2.17. Measurement of NO Production

After 48 h of spleen cells culture (1 × 10^6^ cells/mL) in the presence of SLA (12.5 μg/mL) or 72 h of BMMs infection, culture supernatants were collected and analyzed for their nitrite content. Briefly, 50 μL of supernatant were mixed with an equal volume of Griess reagent (1% sulfanilamide and 0.1% N-1-naphthylethyleme diamine hydrocholide in 50% H_3_PO_4_) and incubated at room temperature for 10 min. Absorbance was measured at 570 nm and the results were expressed in μM of nitrite.

### 2.18. Statistical Analysis

All results are expressed as mean ± standard deviation (s.d.). The statistical analysis was conducted using GraphPad Prism version 6.0 software (San Diego, CA, USA) by applying one-way ANOVA with multiple comparisons Tukey–Kramer post hoc test or two-way ANOVA with multiple comparisons Bonferroni post hoc test, when required. A value of *p* < 0.05 was considered to be significant for all analyses, unless stated otherwise. In the flow cytometry analysis, all antigen-specific cytokine frequencies came after background subtraction of the cytokine frequency of the identically gated population of cells from the same sample in medium alone. 

## 3. Results

### 3.1. In Silico Prediction and Selection of Candidate CTL and HTL Epitopes for the Design of the Multi-Epitope Vaccine 

The T cell epitopes which were incorporated into our experimental vaccine were predicted by computational analyses of the amino acid sequences of the following six previously reported immunoreactive proteins of *L. infantum* parasite: cyclophilin 2, cyclophilin 40, enolase, mitochondrial chaperonin HSP60, dihydrolipoamide dehydrogenase, and one hypothetical protein [15]. Specifically, CTL and HTL epitopes against H2-Dd, H2-Kd, and H2-Ld mouse alleles, and H2-IAd and H2-IEd alleles, respectively, that were recognized as highly scored epitopes were selected. As a result, a total of 10 CTL (Table S1) and 28 HTL epitopes (Table S2) from all six proteins were obtained. Then, the selected epitopes were subjected to a second round of analysis using the IEDB server against HLA class I and II alleles that provide >97% population coverage. On the basis of this analysis, nine CTL and 25 HTL epitopes were selected based on the prediction threshold applied (Tables S3 and S4). Subsequently, the selected epitopes were further evaluated in terms of conservancy against murine and human proteome to exclude homologous epitopes with human and murine proteins. As a result, none of the screened epitopes were found to be homologous with any of the human or murine proteins. Moreover, the conservancy analysis among different *Leishmania* species showed that all the selected epitopes were highly conserved among *L. infantum*, *L. major*, *L. donovani*, *L. mexicana*, and *L. braziliensis* parasites and in some cases were 100% identical (Table S5). Thus, the epitopes with the highest conservancy among *Leishmania* spp. were selected as the candidate vaccine epitopes. Finally, these epitopes were found to have altogether about 97.29% population coverage in the world population, confirming the correctness of epitopes selection for the design of a global vaccine against leishmaniasis (Table 1 and Table 2).

### 3.2. Design and Characterization of the Multi-Epitope Vaccine Construct

**Figure 1:**
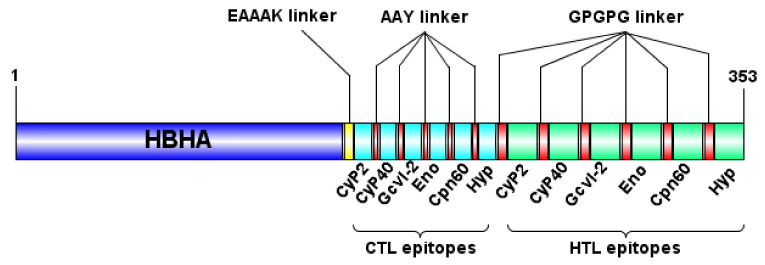
Schematic representation of the designed multi-epitope chimeric vaccine. The vaccine sequence consisted of 353 amino acid residues; the first 159 amino acids are related to the heparin-binding hemagglutinin (HBHA) adjuvant followed by 6 CTL epitopes and 6 HTL epitopes linked together by EAAAK, AAY, and GPGPG linkers, respectively.

The selected CTL and HTL epitopes were used for the design of a multi-epitope vaccine construct. Specifically, CTL epitopes were fused together with the AAY linkers, whereas the GPGPG linkers were used for the fusion of the HTL epitopes to enhance stability and antigen processing. Moreover, in order to enhance the vaccine’s induced immune responses, the 1–159 amino acid sequence obtained from HBHA of *M. tuberculosis* was joined to the vaccine construct at its N-terminal site by the EAAAK linker. The GPGPG linker induce T_H_ responses by keeping conformational dependent immunogenicity of HTL epitopes [41], AAY are the cleavage site of proteasomes creating epitopes appropriate for TAP transporter or other chaperones [42], and the EAAAK linker applied between the HBHA domain and multi-epitope domain separates them providing structural flexibility and preserved bioactivity of the structure [43,44]. Consequently, the final vaccine construct was composed of 353 amino acid residues and consisted of the following three domains: the HBHA adjuvant at the N-terminal site, six CTL epitopes and six HTL epitopes fused together by one EAAAK, and five AAY and five GPGPG linkers (Figure 1). The physicochemical properties of the multi-epitope vaccine construct were determined using the ProtParam server (Table S6). It was found that the molecular weight of the construct was 36.5 kDa with a theoretical isoelectric point of 6.15 (pI) indicating the acidic nature of the protein. Moreover, it was qualified as a stable protein with a low instability index of 31.03, whereas the high aliphatic index of 87.05 indicated protein’s high thermostability (Table S6). The grand average of hydropathicity (GRAVY) was -0.165 which represented its hydrophilic nature. Regarding the allergenicity parameter, the protein vaccine construct was evaluated as non-allergen based on the combined predictions of the AlgPred, Allerdictor, and AllergenFP servers (Table S6). Finally, it was predicted to be an antigenic protein with a probability score of 0.7132 by the VaxiJen server at 0.4 threshold and 0.803170 by ANTIGENpro (Table S6).

### 3.3. Effects of the Multi-Epitope Domain of the Chimeric Protein on Interactions with the TLR4/MD2 Complex

**Figure 2:**
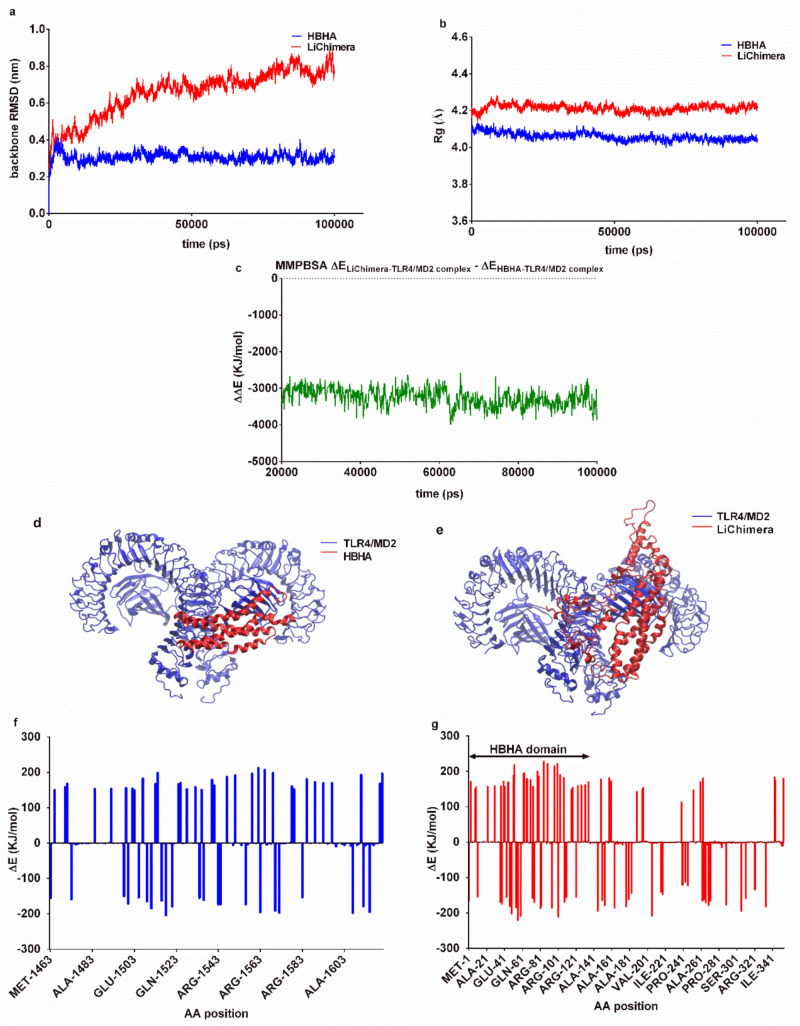
Molecular dynamics of HBHA and LiChimera complexes bound on TLR4/MD2. (**a**) RMSD changes of backbone atoms as compared with the starting structure of (**a**) HBHA and LiChimera complex; (**b**) Radius of gyration (R_g_) of HBHA-TLR4/MD2 and LiChimera-TLR4/MD2 complex during the trajectories indicated that the predicted docking solutions retained their compactness; (**c**) Difference of MMPBSA calculated binding energies between LiChimera and HBHA during the course of the simulations; Backbone representation of (**d**) HBHA-TLR4/MD2 and (**e**) LiChimera-TLR4/MD2 complex; Residue contribution on MMPBSA binding energies of (**f**) HBHA and (**g**) LiChimera on TLR4/MD2 complex.

HBHA-induced immune responses have been proposed to be mediated through interaction with the TLR4 receptor and specifically the MD2 adaptor protein [28]. In order to examine any likely effects of the multi-epitope domain of LiChimera on HBHA interactions with the TLR4/MD2 complex, we compared the binding characteristics of the HBHA and LiChimera molecular models using molecular dynamics simulations. The stability of the best docking structures was assessed through RMSD of backbone atoms and radius of gyration during the obtained trajectories. The TLR4/MD2-HBHA complex stabilized after approximately 50 ns, while the respective LiChimera complex was less stable with the backbone exhibiting limited shifts after 75 ns (Figure 2a). Both complexes retained their compactness, as determined by radius of gyration calculation, with the HBHA complex being more compact as compared with the LiChimera (4.06 ± 0.02 Å versus 4.21 ± 0.02 Å, Figure 2b). The estimation of the MMPBSA binding energies indicated that the LiChimera was interacting stronger with the TLR4/MD2 complex as compared with the HBHA (ΔΔE = −3253 ± 252 KJ/mol, Figure 2c). This was probably due to the wider contact area observed with the TLR4/MD2-LiChimera complex (Figure 2d,e). The analysis of the contribution of each amino acid to binding energy revealed that the non-HBHA domain of the chimeric protein had a positive effect on binding (negative ΔE values), whereas the HBHA domain, although somewhat differently bound, exhibited a similar contribution with the HBHA protein in the respective complex (Figure 2f,g). The above data indicated that the multi-epitope domain in the LiChimera protein would most likely have no negative effects on interactions of the HBHA coiled-coil domain with the TLR4/MD2 receptor.

### 3.4. Recombinant LiChimera Recognized by Canine PBMCs

**Figure 3:**
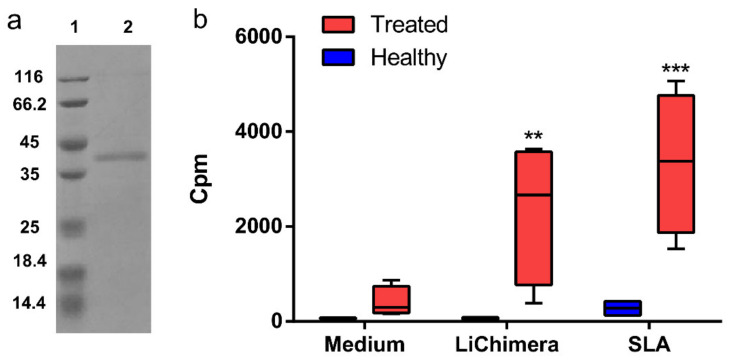
Recombinant LiChimera is antigenic in canine peripheral blood mononuclear cells (PBMCs) from *Leishmania* infected dogs. (**a**) The gene from LiChimera was cloned into pET-30a(+) plasmid and overexpressed in *E. coli* BL21 by isopropyl-β-D-thiogalactopyranoside (IPTG) induction. The recombinant protein was purified by Ni-NTA chromatography, residual endotoxins were removed, and purity was confirmed by SDS-PAGE. Lane 1, molecular weight marker and lane 2, LiChimera; (**b**) PBMCs were isolated from healthy dogs (*n* = 5) and cured from *Leishmania* infected dogs (*n* = 5). PBMCs were stimulated with medium, LiChimera (10 μg/mL), or soluble *Leishmania* antigen (SLA) (10 μg/mL) for 96 h. Antigen-specific proliferation was determined by [^3^H]-thymidine incorporation after another 18 h of culture and expressed as counts per minute (cpm). Bounds of box and whisker plots represent the min-to-max fraction of cpm. The line represents the median, the whiskers show the data range, and the box shows the interquartile range. Significant differences between groups are indicated with asterisks measured by one-way ANOVA and Tukey’s multiple comparison tests. ** *p* < 0.01 and *** *p* < 0.001.

The synthesized 1058-bp DNA which encoded the multi-epitope vaccine was expressed as soluble protein in *E. coli* by IPTG induction. The analyses by SDS-PAGE showed a single band of 37 kDa (Figure 3a). As the multi-epitope domain of the vaccine consisted of epitopes obtained from *Leishmania* proteins recognized exclusively by asymptomatic dogs infected with *L. infantum*, its antigenicity was investigated in the canine PBMCs obtained from the dogs treated against leishmaniasis. The PBMCs from the healthy dogs served as the control group. As shown in Figure 3b, the PBMCs from treated dogs showed significantly higher levels of proliferation as compared with those obtained from healthy ones after stimulation with LiChimera (*p* < 0.01), although in lower levels as compared with SLA (*p* < 0.001) stimulation which served as the positive control. 

### 3.5. Detection of Vaccine Safety

The safety profile of the proposed vaccine was extrapolated by the observation of mice behavior and physiology throughout the study period. There was no morbidity or mortality that occurred in any animal and no skin reactions, such as erythema or edema were found at the site of injection. Animals’ mobility after vaccine injection was normal and they did not display any difference from the PBS control group in group average of body mass and body mass change or food consumption during the study period. In general, all the animals maintained constant body condition throughout the study. In addition, the absolute numbers of splenic populations were comparable among groups (Figure S1). As a conclusion, the vaccine appeared to be well tolerated. 

### 3.6. Characterization of Humoral and Cellular Immune Responses Induced by LiChimera Vaccination in a Murine Experimental Model of Visceral Leishmaniasis

**Figure 4:**
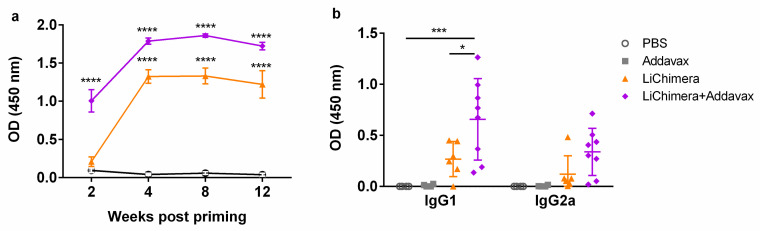
Detection of LiChimera-specific antibodies in LiChimera-vaccinated mice. The BALB/c mice were intramuscularly vaccinated two times at two-week intervals with LiChimera alone or adjuvanted with Addavax. (**a**) Kinetics of the LiChimera-specific serum IgG production on weeks 2, 4, 8, and 12 post priming were determined by ELISA; (**b**) Two weeks post boosting, serum samples were assayed for antigen-specific IgG1 and IgG2a antibodies by ELISA. Data are expressed as means ± s.d. for *n* = 6 (PBS and Addavax) or *n* = 8 mice (LiChimera and LiChimera/Addavax). Significant differences between groups are indicated with asterisks measured by one-way ANOVA and Tukey’s multiple comparison tests. * *p* < 0.05, *** *p* < 0.001, and **** *p* < 0.0001.

LiChimera’s immunogenicity was evaluated in the BALB/c murine experimental model of visceral leishmaniasis. For this reason, BALB/c mice were vaccinated with LiChimera alone or adjuvanted with Addavax (LiChimera/Addavax) intramuscularly two times with two-week intervals. Mice vaccinated with adjuvant alone or PBS served as the control groups. First, the induction of antigen-specific IgG antibody responses was assessed at different time points after priming in all mouse groups. As shown in Figure 4a, LiChimera-specific IgG antibodies were detectable two weeks post priming in LiChimera/Addavax-vaccinated mice and their production reached a significant difference versus both of the control mouse groups (*p* < 0.0001, Figure 4a). On the contrary, low levels of antigen-specific IgG were observed in the LiChimera-vaccinated group at this time point (Figure 4a). The second vaccination boosted IgG levels in the LiChimera/Addavax-vaccinated and also in the LiChimera-vaccinated mouse groups and these were sustained until 12 weeks post priming (*p* < 0.0001, Figure 4a). The investigation of IgG isotypes two weeks post boosting revealed that LiChimera alone elicited predominantly IgG1 antibodies. On the contrary, LiChimera adjuvanted with Addavax induced a mixed response with dominance of IgG1 over IgG2a production (Figure 4b). 

**Figure 5:**
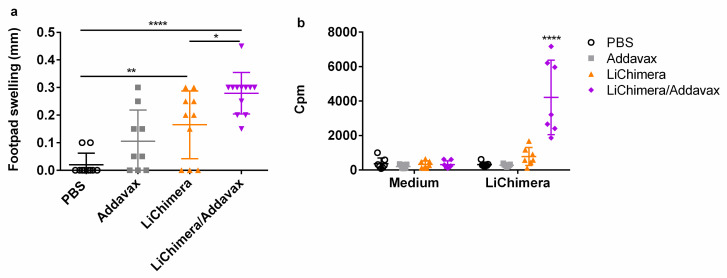
Detection of cellular immune responses following LiChimera vaccination. The BALB/c mice were intramuscularly vaccinated two times at two-week intervals with LiChimera alone or adjuvanted with Addavax. (**a**) Delayed-type hypersensitivity (DTH) responses were evaluated on day 10 post boosting by measuring the difference between the thickness of the test and control footpads at 24 h post injection; (**b**) Spleens were collected two weeks post boosting and splenocytes were stimulated in vitro with LiChimera (2.5 µg/mL) for 72 h. Antigen-specific splenocyte proliferation was determined by [^3^H]-thymidine incorporation after another 18 h of culture and expressed as counts per minute (cpm). Data are expressed as means ± s.d. for *n* = 6 (PBS and Addavax) or *n* = 8 mice (LiChimera and LiChimera/Addavax). Significant differences between groups are indicated with asterisks measured by one-way ANOVA and Tukey’s multiple comparison tests. * *p* < 0.05, ** *p* < 0.01, and **** *p* < 0.0001.

Second, the induction of LiChimera-specific T cell responses was also evaluated, since protection against parasites is based in the induction of strong cellular responses. Initially, the DTH responses against SLA were investigated 10 days post boosting as an indicator of vaccination-induced antigen-specific cellular mediated immune (CMI) responses. Interestingly, the LiChimera-vaccinated mice showed significant footpad swelling (*p* < 0.01) indicating antigen’s immunogenicity (Figure 5a). The DTH response was enhanced when the LiChimera was injected in the presence of Addavax as compared with the LiChimera-vaccinated mice (*p* < 0.05, Figure 5a). This was followed by strong proliferative responses after ex vivo stimulation with LiChimera as compared with the PBS and adjuvant control groups (*p* < 0.001, Figure 5b). On the contrary, the LiChimera-vaccinated mice displayed a small not significant response towards antigen (Figure 5b). 

### 3.7. BMDCs Differentially Pulsed with LiChimera Elicit Strong Antigen-Specific CD4^+^ T Cell Responses 

**Figure 6:**
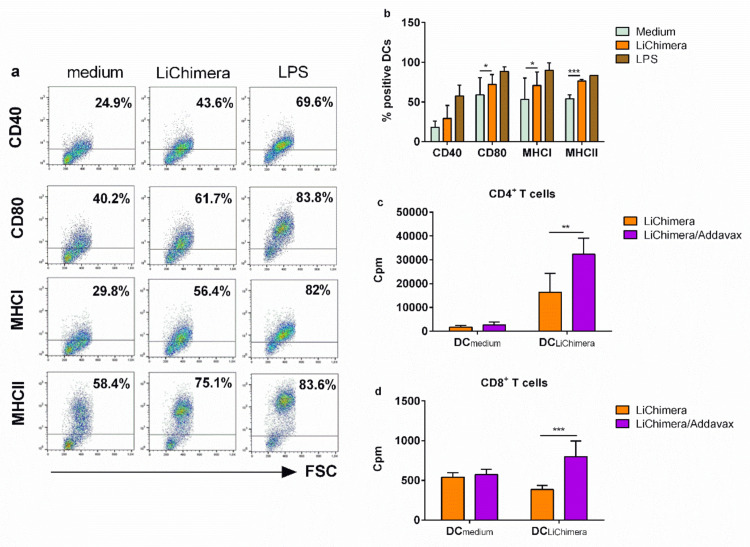
In vitro proliferation of CD4^+^ and CD8^+^ T cells in response to LiChimera-pulsed bone marrow-derived dendritic cells (BMDCs). BMDCs were pulsed with LiChimera for 24 h and expression of CD40, CD80, MHCI, and MHCII was detected with flow cytometry. The BMDCs pulsed with medium alone or lipopolysaccharide (LPS) served as control groups. (**a**) Representative flow cytometry dot plots are shown from three independent experiments; (**b**) Percentage of BMDCs expressing CD40, CD80, MHCI, and MHCII molecules; (**c**,**d**) BMDCs were stimulated with LiChimera for 18 h. BMDCs stimulated with medium alone served as the control. After 18 h, 2 × 10^4^ BMDCs were cocultured with 1 × 10^5^ CD4^+^ T (c) or CD8^+^ T (**d**) cells isolated from mouse vaccinated with LiChimera or LiChimera/Addavax, for 72 h. T cell proliferation was determined by [^3^H]-thymidine incorporation after another 18 h of culture and expressed as counts per minute (cpm). Data are expressed as triplicate means ± s.d. Significant differences between groups are indicated with asterisks measured by one-way ANOVA and Tukey’s multiple comparison tests. * *p* < 0.05, ** *p* < 0.01, and *** *p* < 0.001.

It has been shown that activation of TLR pathways through ligation with their respective ligands on DCs surface induces their phenotypic maturation characterized by increased surface expression of co-stimulatory molecules. This ultimately leads to the generation of strong adaptive immune responses through interaction with T cells. For that reason, the effect of LiChimera on the BMDCs was assessed, since it contains a TLR4/MD2-interacting domain based on the above in silico analysis. As shown in Figure 6, DCs stimulated with LiChimera acquired a mature phenotype characterized by enhancement of CD40 (n.s) and CD80 (*p* < 0.05) co-stimulatory molecules expression as compared with untreated DCs (Figure 6a,b). Notably, the MHC class I (*p* < 0.05) and the MHC class II (*p* < 0.01) expression were also significantly increased (Figure 6a,b). However, their levels were not as high as those detected by lipopolysaccharide (LPS) stimulation, confirming the absence of endotoxin contamination during LiChimera isolation (Figure 6a,b). To further determine whether LiChimera-treated DCs were able to generate strong T cell-mediated immune responses through MHCII and MHCI antigen presentation, we examined CD4^+^ and CD8^+^ T cells proliferation using T cells from mice that were previously immunized with LiChimera alone or adjuvanted with Addavax. T cells were coincubated with LiChimera-pulsed DCs or DCs incubated with medium alone. According to results, LiChimera-pulsed DCs were able to present antigen to CD4^+^ T cells obtained from both vaccinated mice groups (Figure 6c). However, CD4^+^ T cells obtained from LiChimera/Addavax-vaccinated mice responded at higher levels as compared with the CD4^+^ T cells from the LiChimera-vaccinated mice (*p* < 0.01), indicating that the intensity of cellular responses was influenced by the presence of Addavax (Figure 6c). The LiChimera-pulsed DCs were capable of inducing CD8^+^ T cells proliferation observed in the LiChimera/Addavax mouse group only (*p* < 0.001, Figure 6d). However, the CD8^+^ T cells proliferation levels were lower as compared with those detected in the CD4^+^ T cells (Figure 6c,d). As expected, DCs that were pulsed with medium alone did not exhibit any capacity to induce T cell proliferation. Overall, these results indicate that LiChimera alone could promote DCs maturation efficiently and it is processed mainly through MHCII to CD4^+^ T cells.

### 3.8. LiChimera Adjuvanted with Addavax Induced the Differentiation of Multifunctional T Cells 

**Figure 7:**
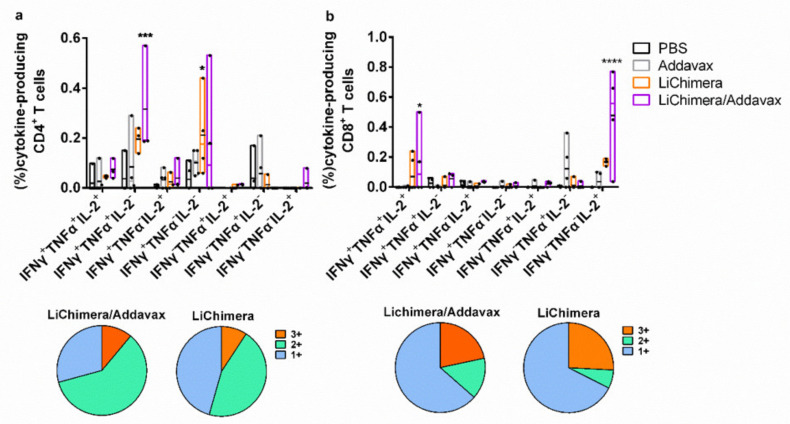

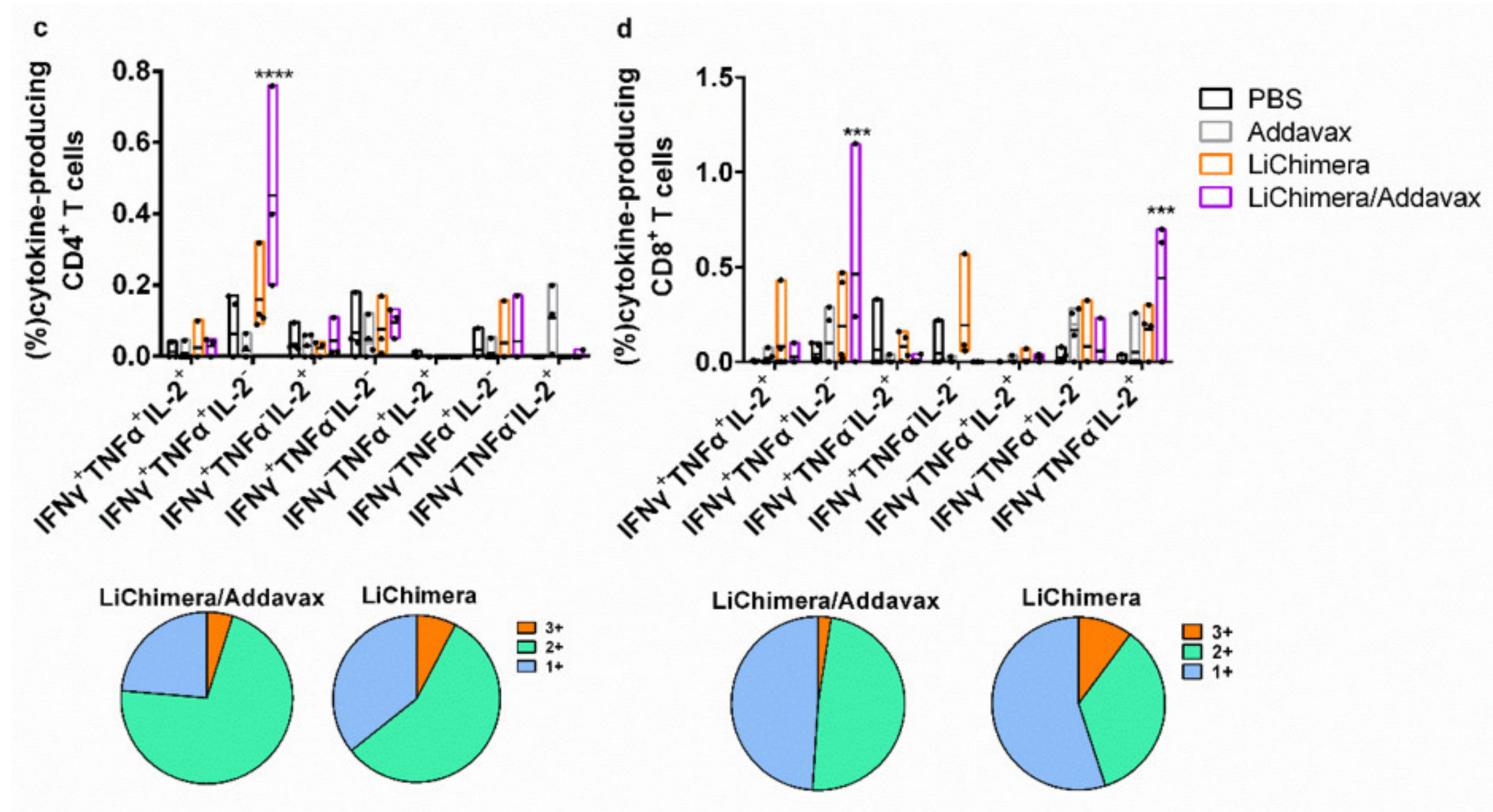
Determination of the multifunctionality of antigen-specific T cell responses following vaccination with LiChimera. The BALB/c mice were vaccinated twice intramuscularly with LiChimera alone or adjuvanted with Addavax. The control groups received PBS or Addavax. Two weeks post boosting, spleen cells were isolated and splenocytes were stimulated in vitro with LiChimera or pools of MHCI- or MHCII-restricted peptides for 12 h. For the antigen-specific CD4^+^ and CD8^+^ T cells, the magnitudes and quality of responses were assessed using flow cytometry multiparametric analysis. Cytokine-producing cells within the CD4^+^ and CD8^+^ T cell populations were divided into seven distinct subpopulations based on their production of these cytokines in any combination after (**a**,**b**) LiChimera, (**c**) MHCII-restricted peptides, and (**d**) MHCI-restricted peptides ex vivo stimulation. The pie charts summarize the fractions of single, double, or triple producers for indicated groups. Flow cytometry results are given as bounds of box and whisker plots. The line represents the median (*n* = 3–4 mice per group), the whiskers show the data range, and the box shows the interquartile range. Significant differences between groups are indicated with asterisks measured by two-way ANOVA and Tukey’s multiple comparison tests. * *p* < 0.05, *** *p* < 0.001, and **** *p* < 0.0001.

**Figure 8:**
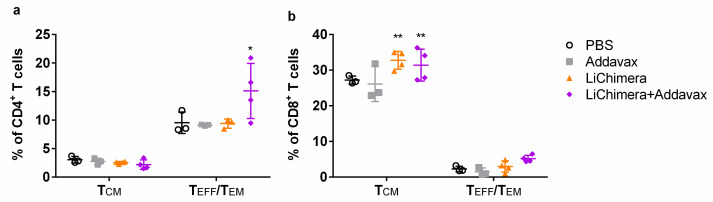
Generation of memory T cell subsets following vaccination with the LiChimera. The BALB/c mice were vaccinated twice intramuscularly with LiChimera alone or adjuvanted with Addavax. The control groups received PBS or Addavax. Two weeks post boosting, spleen cells were isolated and splenocytes were stimulated in vitro with LiChimera for 12 h. The percentages of the LiChimera-specific T_CM_ and T_EFF/EM_ (**a**) CD4^+^ and (**b**) CD8^+^ T cells were assessed by flow cytometry. Data are expressed as means ± s.d. of 3–4 mice per group are shown. Significant differences between groups are indicated with asterisks measured by one-way ANOVA and Tukey’s multiple comparison tests. * *p* < 0.05 and ** *p* < 0.01.

Subsequently, the quality of the LiChimera-specific T cell responses detected in vaccinated mice was assessed by flow cytometry. For this reason, splenocytes were stimulated with LiChimera and also with pools of MHCI- or MHCII-restricted peptides representing the leishmanial amino acid sequence of the multi-epitope domain. Flow cytometry analysis revealed the existence of LiChimera-specific CD4^+^ and CD8^+^ T cells in the mouse groups that received LiChimera adjuvanted with Addavax or LiChimera alone as compared with the PBS and adjuvant mouse groups. On the basis of IFN-γ, TNFα, and IL-2 secretion at the single cell level, both CD4^+^ and CD8^+^ T cells were characterized by high multifunctionality with CD4^+^ T cells (70.8%) having a two-fold higher percentage populations producing two or three cytokines as compared with the CD8^+^ T cells (36.4%) (Figure 5a,b). Specifically, on the one hand, in the LiChimera/Addavax-vaccinated mice, the CD4^+^ T cells had a remarkable percentage of specific IFN-γ^+^TNFα^+^ T cells (51.6%, *p* < 0.01) followed by single IFN-γ^+^ secreting cells (26.3%, *p* < 0.05) (Figure 7a), whereas the CD8^+^ T cells consisted mainly of single IL-2^+^ secreting T cells (61.8%, *p* < 0.001) (Figure 7b) followed by a significant number of triple cytokine (IFN-γ^+^TNFα^+^IL-2^+^: 21.6%, *p* < 0.05) producers (Figure 7b). On the other hand, the LiChimera-vaccinated mice mainly consisted of single-producing IFN-γ^+^ CD4^+^ T cells (*p* < 0.05, Figure 7a). An adaptive immunity phenotype analysis against the pools of MHCI- and MHCII-restricted peptides revealed the existence of peptide-specific CD4^+^, as well as CD8^+^ T cells populations. Importantly, MHCII peptide-stimulated CD4^+^ T cells exhibited a profile similar to the LiChimera-specific CD4^+^ T cells, with 64.2% (*p* < 0.001) of the cell population producing both IFN-γ and TNFα and around 16% being single IFN-γ^+^ cytokine producers in the LiChimera/Addavax-vaccinated mice (Figure 7c). This mouse group also contained MHCI-specific CD8^+^ T cells that consisted of equal numbers of double IFN-γ^+^TNFα^+^ producers (45%, *p* < 0.01) and single IL-2^+^ cytokine producers (43%, *p* < 0.01) (Figure 7d). Interestingly, CD4^+^, as well as CD8^+^ T cells in the LiChimera-vaccinated mice, presented similar profiles to the LiChimera/Addavax-vaccinated mice, although the single producers (IFN-γ^+^CD4^+^ and IL-2^+^CD8^+^ T cells) were enhanced as compared with the triple- and double-cytokine producers (Figure 7c,d). However, these populations did not reach significance as compared with the cells obtained from the control mouse groups. Since memory T cell responses are critical to induce long-term protection against infection, the LiChimera-specific CD4^+^ and CD8^+^ T cells were also evaluated for expression of markers associated with memory cells, i.e., CD44 and CD62L. Detection of those markers allowed the definition of central memory (CD44^+^CD62L^+^, T_CM_) and effector memory T cells (CD44^+^CD62L^-^, T_EFF/EM_) among CD4^+^ and CD8^+^ T cells. As shown in Figure 8, the LiChimera/Addavax vaccination induced the differentiation of a significant larger population of effector memory CD4^+^ (*p* < 0.05) and central memory CD8^+^ T cells (*p* < 0.01) as compared with the PBS and adjuvant control groups. On the contrary, the LiChimera vaccination did not induce the differentiation of any kind of CD4^+^ T cells except from the generation of central memory CD8^+^ T cells (*p* < 0.01). 

### 3.9. Effects of LiChimera on Activation of Macrophages Leishmanicidal Efficacy

**Figure 9:**
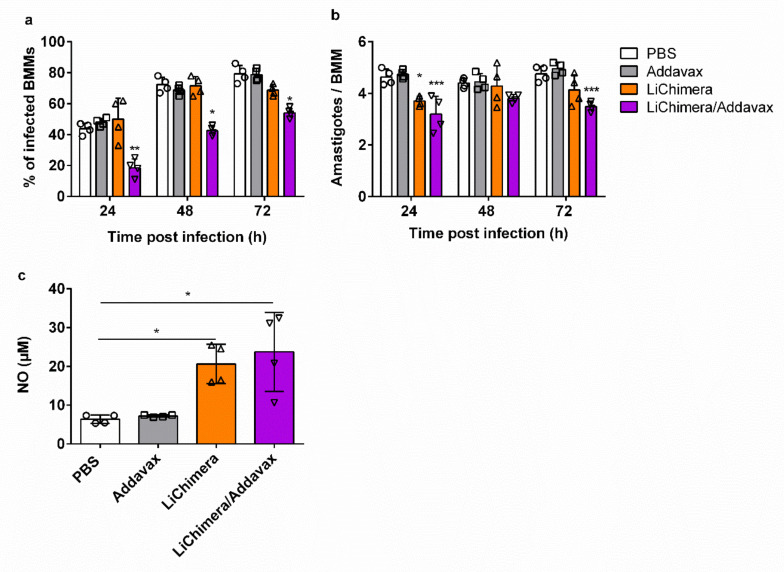
LiChimera vaccination triggers innate responses. The bone marrow-derived macrophages (BMMs) were differentiated from the bone marrow of vaccinated and non-vaccinated mice two weeks post boosting injection followed by in vitro infection with *L. infantum* parasites. At 24, 48, and 72 h post challenge, cells were Giemsa stained for determination of (**a**) percentage of infected BMMs and (**b**) determination of intracellular parasite numbers; (**c**) Detection of NO levels 48 and 72 h post challenge in culture supernatants with Griess reaction. Data are expressed as means ± s.d. of triplicate cultures from *n* = 4 mice per group. Circles, boxes, triangles and reverse triangles represent individual animals from PBS, Addavax, LiChimera or LiChimera/Addavax group, respectively. Significant differences between groups are indicated with asterisks measured by one-way ANOVA and Tukey’s multiple comparison tests. * *p* < 0.05, ** *p* < 0.01, and *** *p* < 0.001.

Considering the fact that macrophages are critical in shaping the innate immunity against *Leishmania* parasite, we also investigated the anti-leishmanial activity of LiChimera vaccine in vitro using bone marrow-derived macrophages. Bone marrow was obtained from vaccinated and non-vaccinated mice two weeks post booster vaccination. Macrophages were differentiated from the bone marrow progenitor cells and were incubated for 72 h after initial in vitro infection with stationary phase *L. infantum*. We observed that the number of infected macrophages from mice vaccinated with LiChimera/Addavax were significantly lower than the PBS and adjuvant controls, as well as the macrophages obtained from the LiChimera-vaccinated mice, in the time period of 24–72 h (Figure 9a). Furthermore, the mean number of amastigotes in the infected macrophages was controlled in groups of mice vaccinated with LiChimera/Addavax as compared with the other mouse groups (Figure 9b). Since the production of NO from macrophages plays a significant role in *Leishmania* parasite killing, we further detected NO levels after 72 h of macrophages’ exposure to parasites. According to the results, the macrophages obtained from the vaccinated mice produced increased levels of NO as compared with the macrophages from both of the control groups (*p* < 0.05, Figure 9c). However, the results indicated that the NO levels were inadequate to resolve *Leishmania* infection in macrophages. This is supported by the finding that macrophages differentiated from LiChimera-vaccinated mice despite the high levels of NO production (Figure 9c), they were not able to restrict infection (Figure 9a,b). Overall, LiChimera effectively triggered innate responses for sustained immunity. 

### 3.10. LiChimera/Addavax Vaccination Protects Against L. infantum Challenge 

**Figure 10:**
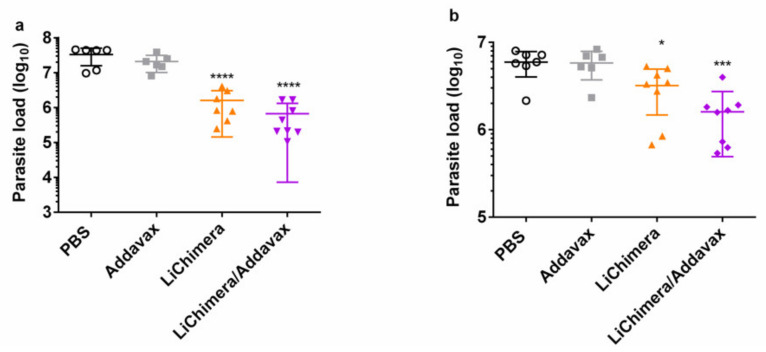

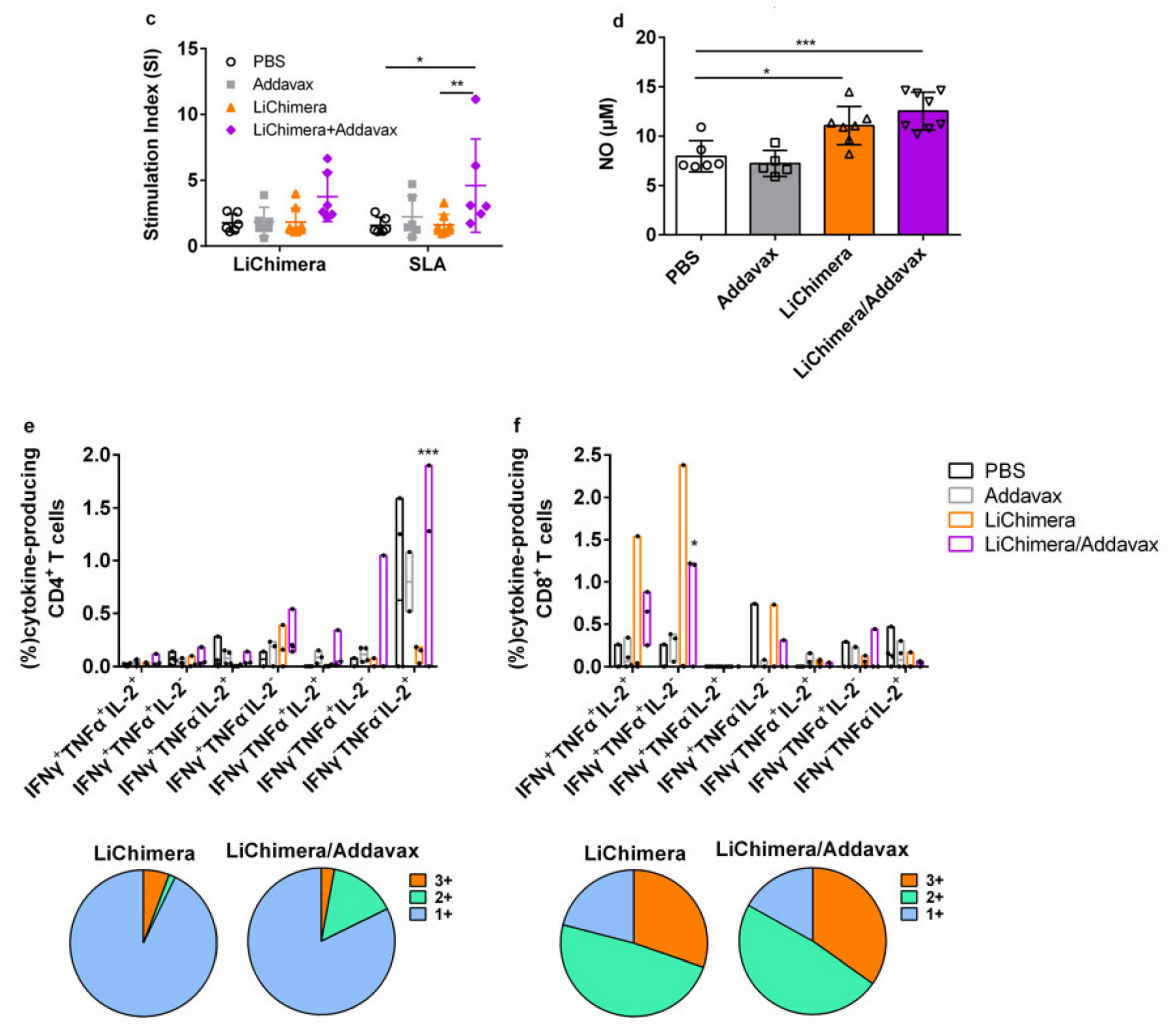
Detection of protective efficacy of the LiChimera vaccination against *L. infantum* challenge and evaluation of the cellular immune responses in short-term vaccinated mice. The BALB/c mice were vaccinated twice intramuscularly with LiChimera alone or adjuvanted with Addavax. The control groups received PBS or Addavax. Two weeks post boosting, mice were challenged with 1 x 10^7^ stationary phase *L. infantum* promastigotes. Eight weeks post challenge the parasite load in liver (**a**) and spleen (**b**) was measured; (**c**) Spleens were collected and splenocytes were stimulated in vitro with LiChimera (2.5 µg/mL) or SLA (12.5 µg/mL) for 72 h. Antigen-specific splenocyte proliferation was determined by [^3^H]-thymidine incorporation after another 18 h of culture and expressed as stimulation index (SI); (**d**) Otherwise, spleen cells were stimulated in vitro with SLA for 48 h and NO production was determined by Griess reaction; Determination of cytokine-producing cells within the (**e**) CD4^+^ and (**f**) CD8^+^ T cell populations. Splenocytes were stimulated in vitro with LiChimera for 12 h and CD4^+^ or CD8^+^ T cell were divided into seven distinct subpopulations based on their production of these cytokines in any combination after the LiChimera stimulation. The pie charts summarize the fractions of single, double, or triple producers for indicated groups. Flow cytometry results are given as bounds of box and whisker plots. The line represents the median (*n* = 3–4 mice per group), the whiskers show the data range, and the box shows the interquartile range. All the other data are expressed as means ± s.d. for *n* = 5–7 (PBS and Addavax) or *n* = 8 (LiChimera and LiChimera/Addavax). Significant differences between groups are indicated with asterisks measured by one-way ANOVA or two-way ANOVA and Tukey’s multiple comparison tests. * *p* < 0.05, ** *p* < 0.01, *** *p* < 0.001, and **** *p* < 0.0001.

The protective efficacy of the LiChimera was evaluated in the experimental model of visceral leishmaniasis in short-term vaccinated mice. Specifically, mice vaccinated with LiChimera alone or adjuvanted with Addavax were infected with stationary phase *L. infantum* promastigotes and protection was assessed through estimation of parasite load in liver and spleen during chronic infection, i.e., eight weeks post challenge. Mice receiving PBS or Addavax alone served as the control infection mouse groups. According to results, the LiChimera/Addavax-vaccinated mice exhibited a significant reduction of parasite load in both liver (98%, *p* < 0.0001, Figure 10a) and spleen (73%, *p* < 0.05, Figure 10b) as compared with the PBS control group. Importantly, the LiChimera/Addavax-vaccinated mice were characterized by higher resistance levels than that afforded by the LiChimera alone in spleen (73% vs. 46%, Figure 10b), whereas in liver, similar levels of protection were detected (Figure 10a). As expected, Addavax alone did not confer any protection against *L. infantum* challenge. Reduction in parasite load in liver was accompanied with a small, not significant, decrease of organ weight in vaccinated mice (Figure S2) which was indicative of parasite restriction. 

Subsequently, an investigation of the phenotype of the cellular immune responses was conducted in the infected mice. Ex vivo splenocyte recall assay revealed the restoration of CMI responses only in the LiChimera/Addavax-vaccinated mice. Specifically, the splenocytes exhibited two-fold and four-fold higher proliferation in response to LiChimera and SLA, respectively, as compared with both of the control groups, as well as the LiChimera mouse group (Figure 10c). Supplementary to this finding, the detection of significant levels of NO produced in response to SLA by splenocytes was obtained from the LiChimera/Addavax-vaccinated mice group (*p* < 0.001), indicating the existence of macrophages with leishmanicidal properties (Figure 10d). Next, the quality of the types of antigen-specific CD4^+^ and CD8^+^ T cells present in the spleens of the infected mice were assessed. According to analysis, the majority of the CD4^+^ T cells in the LiChimera/Addavax-vaccinated mice were shifted towards single IFN-γ^+^, TNFα^+^, and IL-2^+^ cytokine producers (82.4%, Figure 10e) indicating the presence of effector CD4^+^ T cells, whereas the CD8^+^ T cells mainly showed a multifunctional profile represented by IFN-γ^+^TNFα^+^ (47.3%, *p* < 0.05) and IFN-γ^+^TNFα^+^IL-2^+^ (34.8%) cells (Figure 10f). 

### 3.11. LiChimera/Addavax Vaccination Induced Persisting Immunity and Long-Term Protection against L. infantum

**Figure 11:**
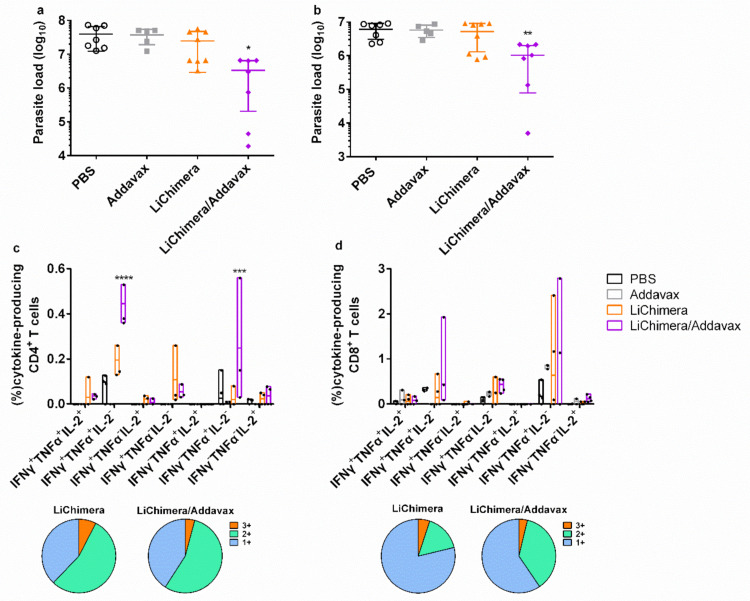
Detection of the protective efficacy of LiChimera vaccination against *L. infantum* challenge and evaluation of the cellular immune responses in long-term vaccinated mice. The BALB/c mice were vaccinated twice intramuscularly with LiChimera alone or adjuvanted with Addavax. The control groups received PBS or Addavax. Ten weeks post boosting, mice were challenged with 1 × 10^7^ stationary phase *L. infantum* promastigotes. Eight weeks post challenge the parasite load in liver (**a**) and spleen (**b**) was measured; Determination of cytokine-producing cells within the (**c**) CD4^+^ and (**d**) CD8^+^ T cell populations. Splenocytes were stimulated in vitro with LiChimera for 12 h and CD4^+^ or CD8^+^ T cell were divided into seven distinct subpopulations based on their production of these cytokines in any combination after LiChimera stimulation. The pie charts summarize the fractions of single, double, or triple producers for indicated groups. Flow cytometry results are given as bounds of box and whisker plots. The line represents the median (*n* = 3–4 mice per group), the whiskers show the data range, and the box shows the interquartile range. All the other data are expressed as means ± s.d. for *n* = 5–7 (PBS and Addavax) or *n* = 8 (LiChimera and LiChimera/Addavax). Significant differences between groups are indicated with asterisks measured by two-way ANOVA and Tukey’s multiple comparison tests. * *p* < 0.05, ** *p* < 0.01, *** *p* < 0.001, and **** *p* < 0.0001.

To evaluate the persistence of LiChimera-induced immunity, vaccinated, as well as the control mice, were also challenged with *L. infantum* 10 weeks post boosting vaccination and protection against parasites was assessed. Mice vaccinated with LiChimera/Addavax maintained high and significant protection against parasite in both liver (88%, *p* < 0.05) and spleen (83%, *p* < 0.01) (Figure 11a,b) as compared with the PBS and adjuvant control groups. On the contrary, the LiChimera-vaccinated mice failed to provide sustainable protection against infection. Specifically, there was only minimal reduction in liver (37%) and spleen (14%) parasite load (Figure 11a,b). To explore the reason for the persistent protection in the LiChimera/Addavax-vaccinated mice, the quality of cellular, as well as humoral responses, was investigated. Interestingly, we continued detecting IFN-γ^+^TNFα^+^ producing cells, as well as single TNFα^+^ and IFN-γ^+^-producing cells, that dominated both of the CD4^+^ and CD8^+^ T cell populations in the LiChimera/Addavax-vaccinated mice (Figure 11c,d). It must be noted that the percentages of double-producing IFN-γ^+^TNFα^+^ populations were two-fold higher in the CD8^+^ T cells (0.82%) than in the CD4^+^ T cells (0.45%) (Figure 11c,d). The LiChimera-vaccinated mice, despite the significant numbers of double producing IFN-γ^+^TNFα^+^ (49.3%) and single-producing IFN-γ^+^CD4^+^ T cells (27%) (Figure 10c), did not exhibit a polyfunctional profile of CD8^+^ T cells (Figure 10d). On the contrary, they were represented by single IL-2^+^, TNFα^+^, and IFN-γ^+^-producing CD8^+^ T cells (92.3%) (Figure 11d). 

**Figure 12:**
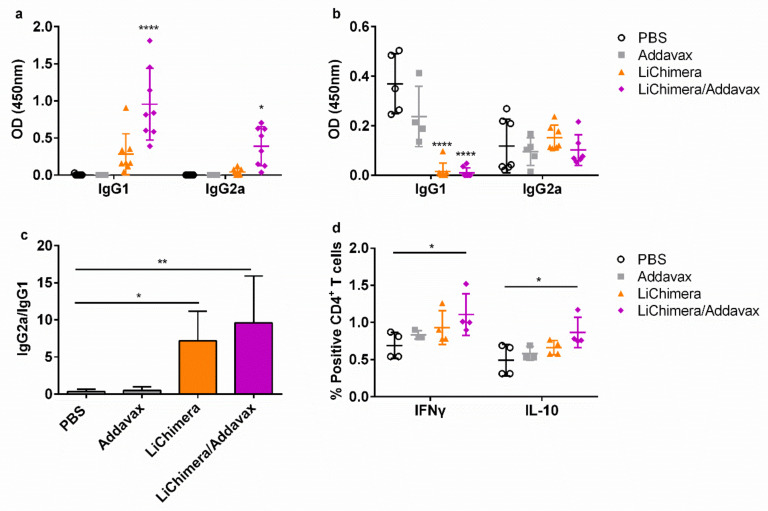
Detection of LiChimera-specific and parasite-specific humoral and cellular responses in long-term vaccinated mice. The BALB/c mice were vaccinated twice intramuscularly with LiChimera alone or adjuvanted with Addavax. The control groups received PBS or Addavax. Ten weeks post boosting, mice were challenged with 1 × 10^7^ stationary phase *L. infantum* promastigotes. Eight weeks post challenge, serum samples were assayed for (**a**) LiChimera-specific and (**b**) parasite-specific IgG1 and IgG2a antibodies by ELISA; (**c**) IgG2a/IgG1 ratio of anti-parasite antibodies. Spleens from each mouse were collected and the obtained splenocytes were stimulated in vitro with SLA (12.5 µg/mL) for 18 h. Determination of IFN-γ and IL-10-producing cells within the CD4^+^ T cell populations was conducted by flow cytometry. (**d**) The ratio of IFN-γ/IL-10 is shown. Data are expressed as means ± s.d. for *n* = 5–7 (PBS and Addavax) or *n* = 8 (LiChimera and LiChimera/Addavax). Significant differences between groups are indicated with asterisks measured by one-way ANOVA. Data are expressed as means ± s.d. for *n* = 6 (PBS and Addavax) or *n* = 8 (LiChimera andLiChimera/Addavax). Significant differences between groups are indicated with asterisks measured by one-way ANOVA and Tukey’s multiple comparison tests. * *p* < 0.05, ** *p* < 0.01, and **** *p* < 0.0001.

Regarding humoral responses, the magnitude of the LiChimera-specific antibodies production remained at high levels and it was independent of the adjuvant used since non-adjuvanted and adjuvanted LiChimera were equally immunogenic (Figure 12a). Importantly, despite the high protection levels, increased LiChimera-specific IgG1 production over IgG2a was still detected (Figure 12a). However, when parasite-specific antibodies were detected, an increased IgG2a/IgG1 ratio was observed in vaccinated mice, indicating the existence of parasite-specific T_H_1 immune responses that have a protective role against infection (Figure 12b,c). To deepen the analysis, the quantification of parasite-specific IFN-γ- and IL-10-producing CD4^+^ T cells was conducted, since the counterbalance of these two cytokines is crucial for the establishment and pathogenesis of VL. According to the results, in the LiChimera/Addavax-vaccinated mice, higher percentages of both populations producing IFN-γ and IL-10 as compared with the control mice were detected (Figure 12d).

### 3.12. Effect Of CD8^+^ T Cell Depletion on Vaccine Efficacy

**Figure 13:**
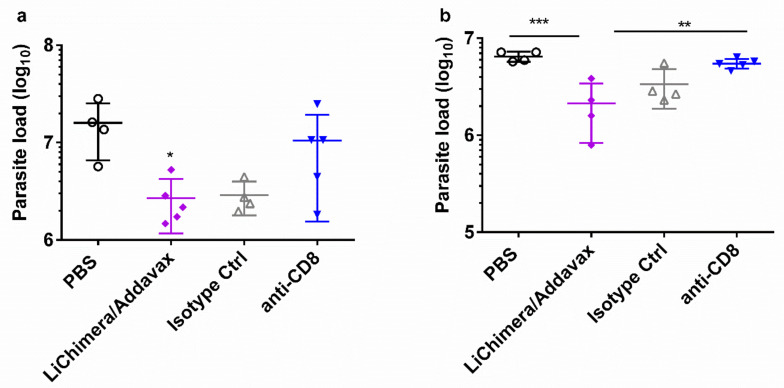
Role of CD8^+^ T cells protection against *L. infantum* in LiChimera/Addavax vaccinated mice. The BALB/c mice were vaccinated twice intramuscularly with LiChimera adjuvanted with Addavax. Two weeks post boosting, mice were challenged with 1 × 10^7^ stationary phase *L. infantum* promastigotes. Mice were treated the day before *L*. *infantum* challenge and again on day 2 using anti-CD8^+^ monoclonal antibody. The control mice were treated with normal rat IgG. As the control for infection, PBS-injected mice were also included. The parasite load in liver (**a**) and spleen (**b**) was measured 8 weeks post challenge. Data are expressed as means ± s.d. for *n* = 4–5 mice per group. Significant differences between groups are indicated with asterisks measured by one-way ANOVA and Tukey’s multiple comparison tests. * *p* < 0.05, ** *p* < 0.01, and *** *p* < 0.001.

To evaluate the relative contribution of the CD8^+^ T cells to the vaccine-elicited protection in mice, the mice were vaccinated with LiChimera and Addavax. Two weeks post boosting mice were challenged with *L. infantum*. Moreover, CD8^+^ T cells were depleted with anti-CD8^+^ monoclonal antibody given on days -1 and +2 relative to the day of *L. infantum* challenge. The control mice were vaccinated with LiChimera adjuvanted with Addavax, and then received purified polyclonal rat IgG isotype antibody or received only PBS. After challenge, all the vaccinated mice receiving the isotype antibody were able to protect spleen and liver. On the contrary, vaccinated mice with depleted CD8^+^ T cells failed to control parasite infection, since only a 15% and 34% reduction of parasite load in spleen and liver relative to the control (PBS) mouse group were detected (Figure 13). Thus, these data implicate that CD8^+^ T cell populations were elicited by vaccination for protection against *L. infantum* challenge.

## 4. Discussion

The development of an effective vaccine against VL has been a difficult task due to the complexity of the parasite life cycle and the mechanisms used by a parasite to evade a host’s immune response. A considerable number of *Leishmania* antigens including TSA, LmSTI1, A2, KMP-11, HASPB1, CPB, p36/LACK, and p45 have been tested as subunit protein or nucleic acid vaccine candidates against VL and have had variable or unreproducible success in inducing protection [45,46,47,48,49,50,51]. The ideal vaccine should be able to induce robust and long-lasting CD4^+^ and CD8^+^ T cell responses. This can be achieved through multi-epitope vaccines due to presentation of a broader range of epitopes to T cells. Indeed, researchers have developed several multicomponent vaccines such as Q protein, Leish-111f, Leish-110f, and KSAC that elicit better protective responses against VL than vaccines based on single antigens [52,53,54,55,56]. Of these, the multicomponent vaccine LEISH-F2/LEISH-111f+MPL-SE was the first defined vaccine candidate to progress to human clinical trials in healthy volunteers in cutaneous leishmaniasis (CL) and mucocutaneous leishmaniasis (ML) patients in Brazil and Peru and healthy subjects in India [57,58,59]. However, only the Q protein entered the market for the development of a vaccine against canine leishmaniasis. 

According to the above data, there is an urgent need for the design of more sophisticated vaccines that could elicit protection against leishmaniasis. With the help of bioinformatics and structural biology tools, vaccinologists have designed numerous novel subunit vaccine candidates [60] aiming to provide better protection [61,62]. In line with this, in the present study we designed a multi-epitope vaccine candidate, named LiChimera, using several bioinfomatic tools and performed preclinical analyses of its immunogenicity and efficacy against challenge with *L. infantum* in the experimental model of VL. LiChimera consisted of six CTL and six HTL epitopes obtained from cyclophilin 2, cyclophilin 40, dihydrolipoamide dehydrogenase, mitochondrial chaperonin HSP60, enolase, and a hypothetical protein amino acid sequence. These proteins were previously recognized as reactive proteins against asymptomatic canine VL sera [15]. It is believed that antigens recognized by treated or asymptomatic patients and dogs are better vaccine candidates since these groups skew their immune responses towards a protective T_H_1 type against infection [6]. 

It has been shown that a simple mixing of an antigen with an adjuvant cannot guarantee that the delivered antigens are recognized and presented by APCs, as the adjuvant and antigen can dissociate after administration. On the contrary, antigen-TLR ligand conjugates help to ensure that both the antigen and adjuvant reach APCs simultaneously, are recognized by surface receptors on APCs, and are internalized together [63]. The TLR agonists can activate DCs, facilitating the uptake of antigens and presentation of antigenic peptides in the context of MHC molecules. Adaptive immunity is, then, established through activation of antigen-specific T lymphocytes and induction of specific humoral and cell-mediated responses. Thus, developing subunit vaccine candidates with built-in TLR agonists has attracted wide attention in vaccine research and development [63]. It has been shown that bacterial factors with lectin-like domains, such as coiled-coil domain, that are responsible for bacteria adherence to cells can prime or control immunity and exert an adjuvant effect [64,65,66]. Thus, their use in subunit vaccines resulted in vaccine improvement due to enhanced processing of the codelivered antigens by the antigen-presenting cells [67,68]. In the case of HBHA, it has been shown that it activated DCs through the TLR4 pathway and, more specifically, by interacting with the MD2 adaptor protein of the TLR4/MD2 complex leading to induction of T_H_1 responses via IFN-γ production [28]. Subsequently, our multi-epitope vaccine was conjugated to the N-terminal sequence of the HBHA from *M. tuberculosis* which is a coiled-coil domain with adhesion properties responsible for bacterial agglutination [69] and also for triggering humoral and cellular immune responses [29]. The whole construct was designed by joining the epitopes and the adjuvant together with the use of EAAAK, AAY, and GPGPG linkers in order to keep the structure of the protein intact, and thus cleaved properly and processed to MHCI and MHCII molecules for delivery to the cell surface [41,42,43]. Moreover, the structural and molecular docking analysis predicted that the N-terminal domain of HBHA interacts with TLR4 though binding to myeloid differentiation factor 2 (MD2) co-receptor and the presence of the multi-epitope domain stabilizes this interaction. This could be explained by the physicochemical properties of the chimeric protein making it ideal for binding to MD2, since MD2 binds more effectively to amphipathic and negatively charged agonists [70].

Validation of the immunoinformatics approach was given by ex vivo stimulation of canine PBMCs and pulsing of murine BMDCs. First, the immunogenic potential of the designed multi-epitope chimeric protein was shown in PBMCs obtained from treated dogs that had been naturally infected with *L. infantum*, suggesting that the included epitopes are being processed effectively and recognized by T cells. Moreover, LiChimera induced BMDCs maturation by upregulating the expression of costimulatory as well as MHCI and MHCII molecules. Mature DCs, characterized by increased surface expression of costimulatory molecules and significant production of pro-inflammatory cytokines, are crucial for the induction of the T_H_ immune responses [71]. As a proof-of-concept, in our study, the LiChimera-pulsed BMDCs could effectively stimulate CD4^+^ T cells and CD8^+^ T cells obtained from mice that had been previously immunized with LiChimera. 

Characterization of the LiChimera-induced immune responses were conducted in the BALB/c mice. The results showed that the mice vaccinated with LiChimera developed antigen-specific cellular and humoral immune responses, which were significantly enhanced when LiChimera was given along with Addavax, a commercial oil-in-water emulsion analogue of the MF59 adjuvant. The main effect of that emulsion was the effective recruitment of APCs to the site of injection followed by their migration to draining lymph nodes for antigens presentation to the CD4^+^ T cells [72,73]. Importantly, we found that bone marrow-derived macrophages from the LiChimera/Addavax-vaccinated mice presented enhanced leishmanicidal capacity as assessed by a decreased percentage of infected cells, which was attributed to increased levels of NO production. This is a significant finding since viscerotropic strains of *Leishmania* are also found in the bone marrow of the host which consisted of hemopoietic stem cells (HSCs) [74]. Thus, it is fundamental to train those cell population to effectively kill *Leishmania* parasites. Limited data exist that consider training of HSCs against *Leishmania* infection. Specifically, it has been shown that mobilization of HSCs during infection with Gram-negative bacteria is mediated through TLRs [75]. In a recent study, it has been shown that BCG vaccination conferred epigenetic changes at the level of HSCs by changing the bone marrow microenvironment leading to enhanced protective capacity against *Mycobacterium* infection [76]. Thus, it is possible that the LiChimera-vaccination could have led to an optimal microenvironment in the bone marrow inducing the proper education of HSCs. It must be noted that the detected leishmanicidal activity in the LiChimera/Addavax-vaccinated mice was not due to exclusive NO production, since macrophages differentiated from the LiChimera-vaccinated mice, despite their high NO levels, were unable to control infection. It has been shown that other factors such as reactive oxygen species (ROS) production and not NO alone, play an important role in the control of leishmaniasis in human monocytes [77]. Thus, the discrepancy in the parasite load among macrophages obtained from the LiChimera/Addavax- and LiChimera-vaccinated mice could be due to the inability of the latter to produce ROS.

Those findings were extended to protection experiments against *L. infantum* challenge where vaccination led to long-term protective immunity in LiChimera/Addavax-vaccinated mice. The spleen cells of protected mice displayed increased parasite-specific proliferation efficacy, as well as increased NO production, demonstrating the existence of protective cellular responses. An assessment of T cells phenotype after vaccination revealed that LiChimera/Addavax-vaccinated mouse group presented increased numbers of antigen-specific multifunctional IFN-γ^+^TNFα^+^IL-2^+^ CD4^+^ and CD8^+^ T cells as compared with the untreated controls. Recent studies have shown that the vaccine-induced generation of both CD4^+^ and CD8^+^ T multifunctional cells was a marker of vaccine efficacy against intracellular pathogens, such as *Leishmania* [78,79,80,81,82,83]. However, the frequency of double-positive IFN-γ^+^TNFα^+^, as well as single-positive IFN-γ^+^ CD4^+^ T cells was higher than that of triple-positive cells. IFN-γ produced by CD4^+^ T effector cells provides resistance against *Leishmania* by activating NO production by macrophages [84,85] and this effect is further enhanced by TNFα [86]. A further evaluation of CD4^+^ T cells phenotype showed that this population consisted of antigen-specific effector CD44^+^ CD4^+^ T cells. Importantly, effector memory, as well as effector CD4^+^ T cells, have been previously characterized as correlates of protection against VL [1,82]. Considering CD8^+^ T cells, we detected significant numbers of single-producing IL-2^+^ CD8^+^ T cells with a central memory phenotype. It has been shown that, in a systemic viral challenge, IL-2-secreting central memory CD8^+^ T cells was the population that conferred improved protection as compared with the effector memory cells [87]. Interestingly, when the LiChimera/Addavax-vaccinated mice were challenged, the quality of immune responses shifted to a more effector-like IFN-γ^+^TNFα^+^ signature for the CD4^+^ T cells, whereas the CD8^+^ T cells kept a low proportion of multifunctional cells. Moreover, the CD8^+^ T cells continued to contain single-positive IL-2 populations even in the long-term challenged mice. The existence of IL-2-producing T cells has been well correlated with effective treatment and the induction of long-term protection against tuberculosis [88,89]. In the case of VL, the capability of CD8^+^ T cells to produce IL-2 depicted the absence of exhausted CD8^+^ T cell populations, whose existence has been shown to be critical for the establishment of disease [90], and thus could be positively correlated with increased protective efficacy against parasite challenge in our study. As shown in previous studies, CD8^+^ T cells play a significant role in the cure of VL, since they are better effector cells for clearance of *Leishmania* [91,92]. This is further supported by our findings in CD8^+^ T cell depletion studies, where the protective potential of the LiChimera/Addavax vaccination was significantly decreased. Surprisingly, we detected a significant production of LiChimera-specific IgG1 antibodies over the IgG2a throughout the study. This finding is supportive of the development and the role of CD8^+^ T cells in protection against *L. infantum* challenge in our experimental model. This observation has also been conducted in previous research studies that showed that increased IgG1 titers had an important role in the generation of memory CD8^+^ T cells contributing to resistance to VL infection through the early development of IL-4 producing T_H_2 cells [48,91,93,94].

Analysis of parasite-specific immune responses in the long-term vaccinated-infected mice showed an increased ratio of IFNγ/IL-10 in CD4^+^ T cells, suggesting polarization of immune responses towards T_H_1 type. This was correlated well with the protected phenotype observed in vaccinated mice. It is well known that IL-10 is strongly involved in progression of experimental and human VL by blocking T_H_1 activation and, consequently, macrophages’ cytotoxic responses by downregulating IFN-γ production, favoring disease establishment [95]. This finding was complemented with an increased anti-parasite IgG2a/IgG1 ratio, since it has been shown that IFN-γ directly regulates IgG2a class switching [96]. However, the production of IL-10, even to lower levels as compared with IFN-γ, suggests a desirable balance in the immune responses, allowing parasite persistence that facilitates protection without causing host damage from the increased production of inflammatory cytokines [97]. 

## 5. Conclusions

The present study demonstrates that a multi-epitope chimeric protein designed using an immunoinformatics approach adjuvanted with Addavax gave promising results showing an appreciable long-term protective response against *L. infantum* challenge in the experimental model of BALB/c mice. Protection was correlated with the induction of the innate and adaptive arms of immune response as assessed by increased activation of the CD4^+^ and CD8^+^ T cells simultaneously producing IFN-γ, TNFα, and IL-2, and induction of microbicidal mechanism of macrophages. Importantly, the CD8^+^ T cells that were elicited by the LiChimera/Addavax vaccination contributed significantly to parasite clearance, and thus to long-term protective immunity. Taken together, the present study supports the view that multi-epitope vaccines can be considered to be effective immunogens when combined with the appropriate adjuvants and have the potential as candidate vaccines against leishmaniasis.

## Figures and Tables

**Table 1:** Selected cytotoxic T lymphocyte (CTL) epitopes among *L*. *infantum* proteins to be a part of the multi-epitope vaccine.

Protein Name	Epitope Sequence	Population Coverage	Conservancy
**CyP2**	126-GPNTNGSQF-134	15.14%	100%
**CyP40**	232-KYAKAVRYL-240	23.33%	100%
**Gcvl-2**	439-EYGASSEDL-447	32.09%	100%
**Enol**	186-VYHALKVII-194	26.18%	100%
**Cpn60**	38-LGPKGRNVI-46	76.14%	100%
**Hyp**	169-LFSCMLTSL-177	21.38%	100%

**Table 2:** Selected helper T lymphocyte (HTL) epitopes among *L*. *infantum* proteins to be a part of the multi-epitope vaccine.

Protein Name	Epitope Sequence	Population Coverage	Conservancy
**CyP2**	173-DRPVKPVKIVASGEL-187	59.52%	100%
**CyP40**	332-SEAKEKVKAQKAKLA-340	71.01%	100%
**Gcvl-2**	19-GGPGGYVAAIKAAQL-33	79.83%	100%
**Enol**	111-GCSMAISKAAAAKAG-125	80.49%	100%
**Cpn60**	27-VTRAVAAVATTLGPK-41	66.37%	100%
**Hyp**	57-VAITEDVALAAVQAV-71	52.49%	100%

## Supplementary Materials

The following are available online at https://www.mdpi.com/2076-393X/8/3/350/s1, Table S1: Predicted CTL epitopes of selected *L. infantum* proteins to be a part of multi-epitope vaccine against murine MHCI, Table S2: Predicted HTL epitopes of selected *L. infantum* proteins to be a part of multi-epitope vaccine against murine MHCII, Table S3: Predicted CTL epitopes of selected *L. infantum* proteins against HLA class I alleles that provide >97% population coverage using IEDB server, Table S4: Predicted HTL epitopes of selected *L. infantum* proteins against HLA class II alleles that provide >97% population coverage using IEDB server, Table S5: Conservancy analysis of the predicted sequences among different *Leishmania* species, Table S6: Analysis of the physicochemical and immunological characteristics of the designed multi-epitope vaccine construct, Figure S1: BALB/c mice were vaccinated twice intramuscularly with LiChimera alone or adjuvanted with Addavax. Control groups received PBS or Addavax. Two weeks post boosting, spleen cells were isolated and total cell numbers were determined for each spleen. Circles, boxes, triangles and reverse triangles represent individual animals from each group and lines represent means. Significant differences between groups are indicated with asterisks measured by one-way ANOVA and Tukey’s multiple comparison tests, Figure S2: Liver and spleen weights of short-term vaccinated mice 2 months post *L. infantum* challenge. Circles, boxes, triangles and reverse triangles represent individual animals from each group and lines represent means.
