# Supplementary material for: A Canine-Directed Chimeric Multi-Epitope Vaccine Induced Protective Immune Responses in BALB/c Mice Infected with Leishmania infantum
Source: Vaccines (Basel). 2020 Jun 30;8(3):350. doi: 10.3390/vaccines8030350 (PMC7563305; doi:10.3390/vaccines8030350)
Supplement: Supplementary file 1 [file vaccines-08-00350-s001.pdf]

Table S1. Predicted CTL epitopes of selected *L. infantum* proteins to be part of ~~mutli~~multi-epitope ~~chimeric protein~~vaccine against murine MHCI.

No	Protein Name	Epitope Sequence	IEDB	SYFPEITHI	MHCI
1	**CyP2**	126-GPNTNGSQF-134	0.6	21	H2-Ld
2	**CyP40**	4-TYCYLDITI-12	0.5	23	H2-Kd
3		232-KYAKAVRYL-240	0.2	26	H2-Kd
4		119-YYKGEGSFV-127	0.8	24	H2-Kd
5	**Gcvl-2**	352-NYNVIPGVI-360	0.5	21	H2-Kd
6		439-EYGASSEDL-447	0.7	22	H2-Kd
7	**Enol**	186-VYHALKVII-194	0.5	23	H2-Kd
8	**Cpn60**	38-LGPKGRNVI-46	0.3	-	H2-Dd
9	**Hyp**	92-ESQKSLYEF-100	1	22	H2-Ld
10		169-LFSCMLTSL-177	0.8	22	H2-Ld

Table S2. Predicted HTL epitopes of selected *L. infantum* proteins to be part of multi~~tli~~-epitope ~~chimeric protein~~vaccine against murine MHCII.

No	Protein Name	Epitope Sequence	IEDB	SYFPEITHI	NetMHCII
1		1-MRVVAVLAVVLCALS-15	1.25	-	0.8
2	**CyP2**	2-RVVAVLAVVLCALSF-16	8.87	27	-
3		3-VVAVLAVVLCALSFL-17	18.002	23	-

4		4-VAVLAVVLCALSFLN-18	17.82	22	-
5		19-VAAEPEVTAKVYFDV-33	19.44	23	-
6		173-DRPVKPVKIVASGEL-187	10.73	21	-
7	**CyP40**	296-RRGTAALKAGDADGA-310	0.31	32	426.8
8		332-SEAKEKVKAQKAKLA-346	0.16	28	299.9
9	**Gcvl-2**	19-GGPGGYVAAIKAAQL-33	0.88	28	447.8
10		84-ENVTMDVSAMQAQKA-98	0.05	34	88.3
11		87-TMDVSAMQAQKAKGV-101	0.07	22	96.7
12		456-TMSEAVKEACMACFA-470	5.09	23	219.1
13	**Enol**	111-GCSMAISKAAAAKAG-125	0.07	22	156.4
14		113-SMAISKAAAAKAGVP-127	0.48	29	320.4
15		300-DEFSAITMALAGKAQ-314	1.88	20	268.4
16	**Cpn60**	19-ARRSMQKGVTRAVAA-33	1.39	22	-
17		27-VTRAVAAVATTLGPK-41	1.65	25	-
18		42-GRNVIIEQAYGAPKI-56	6.36	21	-
19		220-AELEDALVLMSAKKI-234	5.79	27	-
20		272-NKLQGKLKVCCVKAP-286	9.55	22	-
21		374-KLKERLAKLSGGVAV-388	4.13	23	-
22		510-PTRVVRVAISDATSV-524	3.91	21	-
23	**Hyp**	57-VAITEDVALAAVQAV-71	1.67	26	159.0
24		58-AITEDVALAAVQAVG-72	0.96	21	184.3
25		59-ITEDVALAAVQAVGL-73	0.67	29	221.7
26		172-CMLTSLAATVIARDL-186	2.54	22	236.2

27		278-RRAAEELALDGVTKD-292	6.28	21	494.9
28		470-PPRNKAVAVKRLQQV-484	3.27	22	259.2

Table S3. Predicted CTL epitopes of selected *L. infantum* proteins against HLA class I alleles that provide >97% population coverage using IEDB server.

No	Protein Name	Epitope Sequence	HLA class I
1	**CyP2**	126-GPNTNGSQF-134	HLA-B*07:02, HLA-B*35:01, HLA-B*53:01, HLA-B*44:02
2	**CyP40**	4-TYCYLDITI-12	HLA-A*23:01, HLA-A*24:02
3		232-KYAKAVRYL-240	HLA-A*24:02, HLA-A*23:01, HLA-A*30:01, HLA-A*31:01, HLA-B*07:02
4	**Gcvl-2**	119-YYKGEGSFV-127	HLA-A*24:02, HLA-A*23:01, HLA-A*30:01
5		352-NYNVIPGVI-360	HLA-A*24:02, HLA-A*23:01, HLA-B*51:01
6		439-EYGASSEDL-447	HLA-A*24:02, HLA-A*23:01
7	**Enol**	186-VYHALKVII-194	HLA-A*24:02, HLA-A*23:01, HLA-B*51:01, HLA-A*32:01
8	**Cpn60**	38-LGPKGRNVI-46	-
9	**Hyp**	92-ESQKSLYEF-100	HLA-A*01:01, HLA-A*26:01, HLA-A*23:01, HLA-B*58:01, HLA-A*24:02, HLA-B*15:01, HLA-B*57:01, HLA-B*35:01
10		169-LFSCMLTSL-177	HLA-A*02:03, HLA-A*02:06, HLA-B*08:01, HLA-A*23:01, HLA-A*24:02, HLA-B*40:01

Table S4. Predicted HTL epitopes of selected *L. infantum* proteins against HLA class II alleles that provide >97% population coverage using IEDB server.

No	Protein Name	Epitope Sequence	HLA class II
1	**CyP2**	1-MRVVAVLAVVLCALS-15	HLA-DRB1*15:01, HLA-DQA1*01:02/DQB1*06:02, HLA-DRB1*01:01, HLA-DRB1*08:02, HLA-DQA1*05:01/DQB1*03:01, HLA-DPA1*03:01/DPB1*04:02, HLA-DQA1*05:01/DQB1*02:01
2		2-RVVAVLAVVLCALSF-16	HLA-DQA1*01:02/DQB1*06:02, HLA-DRB1*01:01, HLA-DPA1*03:01/DPB1*04:02
3		3-VVAVLAVVLCALSFL-17	HLA-DRB1*01:01, HLA-DQA1*01:02/DQB1*06:02, HLA-DPA1*02:01/DPB1*05:01, HLA-DRB1*15:01, HLA-DQA1*05:01/DQB1*02:01, HLA-DPA1*03:01/DPB1*04:02, HLA-DPA1*02:01/DPB1*01:01
4		4-VAVLAVVLCALSFLN-18	HLA-DPA1*02:01/DPB1*05:01, HLA-DPA1*03:01/DPB1*04:02, HLA-DPA1*01:03/DPB1*02:01
5		19-VAAEPEVTAKVYFDV-33	-
6		173-DRPVKPVKIVASGEL-187	HLA-DRB1*08:02, HLA-DRB1*07:01, HLA-DRB1*15:01
7	**CyP40**	296-RRGTAALKAGDADGA-310	HLA-DQA1*05:01/DQB1*03:01
8		332-SEAKEKVKAQKAKLA-346	HLA-DRB1*09:01, HLA-DRB4*01:01
9	**Gcvl-2**	19-GGPGGYVAAIKAAQL-33	HLA-DRB1*08:02, HLA-DRB1*11:01, HLA-DQA1*05:01/DQB1*03:01, HLA-DRB1*09:01, HLA-DPA1*02:01/DPB1*14:01, HLA-DRB1*01:01, HLA-DRB5*01:01, HLA-DQA1*04:01/DQB1*04:02, HLA-DRB1*07:01, HLA-DRB1*15:01
10		84-ENVTMDVSAMQAQKA-98	HLA-DQA1*01:02/DQB1*06:02, HLA-DRB1*03:01, HLA-DRB1*09:01, HLA-DQA1*04:01/DQB1*04:02, HLA-DRB1*04:01, HLA-DRB1*01:01
11		87-TMDVSAMQAQKAKGV-101	HLA-DQA1*01:02/DQB1*06:02, HLA-DRB1*09:01, HLA-DRB1*01:01
12		456-TMSEAVKEACMACFA-470	-
13	**Enol**	111-GCSMAISKAAAAKAG-125	HLA-DRB1*09:01, HLA-DQA1*05:01/DQB1*03:01, HLA-DPA1*02:01/DPB1*14:01, HLA-DRB1*08:02, HLA-DQA1*01:02/DQB1*06:02, HLA-DRB3*02:02, HLA-DRB1*01:01, HLA-DRB1*07:01, HLA-DRB5*01:01

14		113-SMAISKAAAAKAGVP-127	HLA-DQA1*05:01/DQB1*03:01, HLA-DRB1*09:01, HLA-DPA1*02:01/DPB1*14:01, HLA-DRB1*08:02, HLA-DQA1*01:02/DQB1*06:02, HLA-DRB3*02:02, HLA-DRB1*01:01, HLA-DRB1*07:01, HLA-DRB5*01:01
15		300-DEFSAITMALAGKAQ-314	HLA-DQA1*01:02/DQB1*06:02, HLA-DPA1*02:01/DPB1*14:01, HLA-DRB1*09:01, HLA-DRB1*08:02, HLA-DRB1*04:01, HLA-DRB1*01:01, HLA-DQA1*05:01/DQB1*03:01, HLA-DQA1*04:01/DQB1*04:02, HLA-DRB1*07:01
16	**Cpn60**	19-ARRSMQKGVTRAVAA-33	HLA-DPA1*02:01/DPB1*14:01
17		27-VTRAVAAVATTLGPK-41	HLA-DQA1*05:01/DQB1*03:01, HLA-DPA1*02:01/DPB1*14:01, HLA-DRB1*08:02, HLA-DQA1*01:02/DQB1*06:02
18		42-GRNVIIEQAYGAPKI-56	HLA-DRB5*01:01
19		220-AELEDALVLMSAKKI-234	HLA-DRB5*01:01, HLA-DPA1*02:01/DPB1*05:01, HLA-DQA1*01:02/DQB1*06:02, HLA-DRB1*13:02, HLA-DRB1*15:01, HLA-DRB1*11:01, HLA-DRB1*01:01, HLA-DPA1*02:01/DPB1*14:01, HLA-DRB1*04:01, HLA-DRB1*12:01
20		272-NKLQGKLKVCCVKAP-286	HLA-DRB1*12:01, HLA-DRB1*01:01
21		374-KLKERLAKLSGGVAV-388	-
22		510-PTRVVRVAISDATSV-524	HLA-DRB1*08:02, HLA-DPA1*02:01/DPB1*14:01, HLA-DRB4*01:01
23	**Hyp**	57-VAITEDVALAAVQAV-71	HLA-DQA1*04:01/DQB1*04:02, HLA-DRB1*03:01, HLA-DQA1*03:01/DQB1*03:02, HLA-DQA1*01:02/DQB1*06:02, HLA-DRB1*13:02
24		58-AITEDVALAAVQAVG-72	HLA-DQA1*04:01/DQB1*04:02, HLA-DQA1*03:01/DQB1*03:02, HLA-DQA1*01:02/DQB1*06:02, HLA-DRB1*03:01, HLA-DQA1*05:01/DQB1*03:01
25		59-ITEDVALAAVQAVGL-73	HLA-DQA1*04:01/DQB1*04:02, HLA-DQA1*03:01/DQB1*03:02, HLA-DRB1*09:01, HLA-DQA1*01:02/DQB1*06:02, HLA-DQA1*05:01/DQB1*03:01, HLA-DRB1*07:01, HLA-DRB1*01:01
26		172-CMLTSLAATVIARDL-186	HLA-DRB1*01:01, HLA-DQA1*01:02/DQB1*06:02, HLA-DPA1*02:01/DPB1*14:01, HLA-DQA1*04:01/DQB1*04:02, HLA-DRB1*09:01, HLA-DQA1*05:01/DQB1*03:01, HLA-DPA1*02:01/DPB1*01:01, HLA-DRB1*12:01, HLA-DPA1*03:01/DPB1*04:02, HLA-DQA1*03:01/DQB1*03:02, HLA-DRB1*07:01
27		278-RRAAEELALDGVTKD-292	HLA-DRB1*03:01
28		470-PPRNKAVAVKRLQQV-484	HLA-DRB4*01:01, HLA-DRB1*11:01

Table S5. Conservancy analysis of the predicted sequences among different *Leishmania* species.

No	Protein Name	Epitope Sequence	*L. major*	*L. donovani*	*L. mexicana*	*L. braziliensis*
1	**CyP2**	126-GPNTNGSQF-134	100%	100%	100%	n.d.
2		173-DRPVKPVKIVASGEL-187	93.3%	100%	93.3%	n.d.
3	**CyP40**	232-KYAKAVRYL-240	100%	100%	100%	88.89%
4		332-SEAKEKVKAQKAKLA-346	93.3%	100%	93.3%	93.3%
5	**Gcvl-2**	439-EYGASSEDL-447	100%	100%	n.d.	88.89%
6		19-GGPGGYVAAIKAAQL-33	100%	100%	n.d.	100%
7	**Enol**	186-VYHALKVII-194	100%	100%	n.d.	88.89%
8		111-GCSMAISKAAAAKAG-125	100%	100%	n.d.	93.3%
9	**Cpn60**	38-LGPKGRNVI-46	100%	100%	100%	100%
10		27-VTRAVAAVATTLGPK-41	100%	100%	100%	100%
11	**Hyp**	169-LFSCMLTSL-177	100%	100%	100%	93.3%
12		57-VAITEDVALAAVQAV-71	88.89%	100%	100%	93.3%

Table S6. Analysis of the physicochemical and immunological characteristics of the designed multi-epitope ~~protein~~ vaccine construct.

**Physicochemical properties**	
Molecular weight	36458.35
Theoretical pI	6.15
Instability index	31.03
Aliphatic index	87.85
GRAVY	-0.165
Number of amino acids	353
Total no. of negatively charged residues (Asp + Glu)	42
Total no. of positively charged residues (Arg + Lys)	41
Solubility/SOLpro	Soluble/ probability 0.96
**Allergenicity**	
AlgPred	Non-allergen
Allerdictor	Non-allergen
AllergenFP	Probable non-allergen
**Antigenicity**	
Vaxigen	0.7132
ANTIGENpro	0.803170

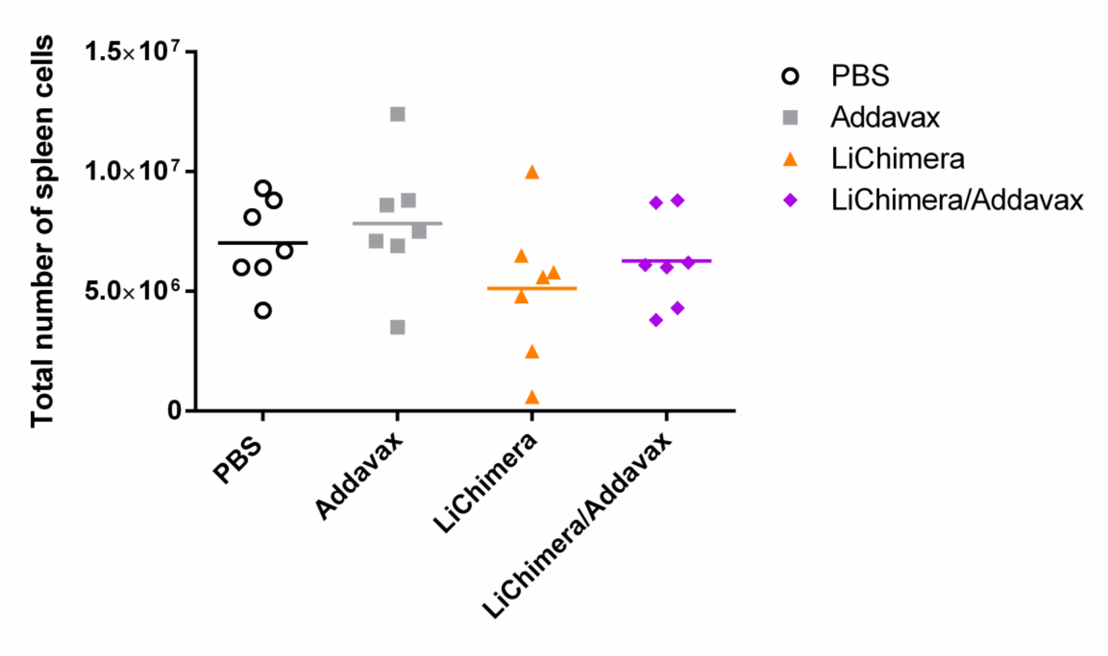

Figure S1. BALB/c mice were vaccinated twice intramuscularly with LiChimera alone or adjuvanted with Addavax. Control groups received PBS or Addavax. Two weeks post boosting, spleen cells were isolated and total cell numbers were determined for each spleen. ~~Dots~~ Circles, boxes, triangles and reverse triangles represent individual animals from each group and lines represent means. Significant differences between groups are indicated with asterisks measured by one-way ANOVA and Tukey's multiple comparison tests.

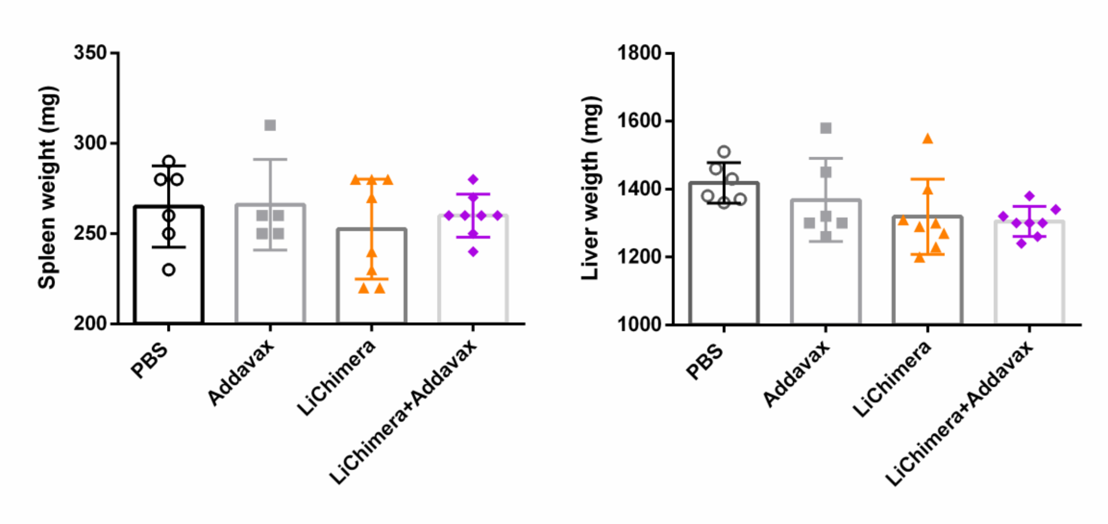

Figure S2. Liver and spleen weights of short-term vaccinated mice 2 months post *L. infantum* challenge. Circles, boxes, triangles and reverse triangles ~~Dots~~ represent individual animals from each group and lines represent means.
